# Combination of ferroptosis and pyroptosis dual induction by triptolide nano-MOFs for immunotherapy of Melanoma

**DOI:** 10.1186/s12951-023-02146-0

**Published:** 2023-10-19

**Authors:** Shengmei Wang, Qiuyan Guo, Rubing Xu, Peng Lin, Guoyan Deng, Xinhua Xia

**Affiliations:** 1 School of Pharmacy, Hunan University of Chinese Medicine, Changsha, 410208 Hunan China; 2 The First Hospital of Hunan University of Chinese Medicine, Changsha, 410007 Hunan China

**Keywords:** Triptolide, Metal-organic framework, Fe^3+^, Ferroptosis, Pyroptosis, Tumor immunotherapy

## Abstract

Immunotherapy has good potential to eradicate tumors in the long term. However, due to the low immunogenicity of tumor cells, current cancer immunotherapies are not effective. To address this limitation, we constructed a BSA-FA functionalized iron-containing metal-organic framework (TPL@TFBF) that triggers a potent systemic anti-tumor immune response by inducing ferroptosis and pyroptosis in tumor cells and releasing large quantities of damage-associated molecular patterns (DAMPs) to induce immunogenicity, and showing excellent efficacy against melanoma lung metastases in vivo. This nanoplatform forms a metal-organic framework through the coordination between tannic acid (TA) and Fe^3+^ and is then loaded with triptolide (TPL), which is coated with FA-modified BSA. The nanoparticles target melanoma cells by FA modification, releasing TPL, Fe^3+^ and TA. Fe^3+^ is reduced to Fe^2+^ by TA, triggering the Fenton reaction and resulting in ROS production. Moreover, TPL increases the production of intracellular ROS by inhibiting the expression of nuclear factor erythroid-2 related factor (Nrf2). Such simultaneous amplification of intracellular ROS induces the cells to undergo ferroptosis and pyroptosis, releasing large amounts of DAMPs, which stimulate antigen presentation of dendritic cells (DCs) and the proliferation of cytotoxic T lymphocytes (CD4+/CD8 + T cells) to inhibit tumor and lung metastasis. In addition, combining nanoparticle treatment with immune checkpoint blockade (ICB) further inhibits melanoma growth. This work provides a new strategy for tumor immunotherapy based on various combinations of cell death mechanisms.

**Supplementary Information:**

The online version contains supplementary material available at 10.1186/s12951-023-02146-0.

## Introduction

Malignant melanoma is a kind of malignant tumor originating from melanocytes [1], which is easy to metastasize to lung, lymph nodes, brain, liver and other organs, among which lung is the most common site of metastasis [2, 3]. Once metastasis occurs, it leads to a very poor prognosis, limited therapeutic benefit, and is the leading cause of death from malignant melanoma [4]. Early definitive diagnosis and radical surgical resection can dramatically improve the prognosis and survival of malignant melanoma. However, there are still significant diagnostic barriers. For example, common nevi and other benign pigmented lesions reduce the predictive value of a positive skin biopsy in melanoma patients. Pathologic diagnosis remains the gold standard for melanoma diagnosis, however, it is sometimes challenging, lacking clear molecular diagnostic and prognostic stratification factors, and lacking objective, highly reproducible criteria that apply to all melanomas. As a result, most patients with malignant melanoma are advanced by the time of definitive diagnosis, and the effect of surgical treatment is not ideal, leading to a very poor prognosis. Chemotherapy and radiation therapy were introduced as treatments for melanoma, both of which kill cancer cells by inducing apoptosis [5].Unfortunately, drug resistance and side effects hindered their effectiveness, with less than 10% of patients with advanced melanoma surviving for more than a few years. The advent of targeted therapies has certainly improved treatment outcomes, however, the disadvantages of drug resistance, short duration of response, and off-target effects result in only about 33% of advanced patients benefiting from 5-year overall survival [6]. Thus, it is necessary to develop innovative and effective cancer treatments that significantly improve treatment outcomes and reduce toxic side effects.

Cancer immunotherapy has received much attention for its ability to activate the body’s own natural defences to identify, attack and eradicate cancer cells [7]. Currently, innovative immunotherapies involving immune checkpoint blockade (ICB) strategies [8], chimeric antigen receptor T-cell engineering therapies [9] and cancer vaccines [10] have yielded some exciting clinical results. In addition, combining immunotherapy with nanotechnology can further enhance the therapeutic effect [11, 12]. However, despite the potential of immunotherapy to eradicate tumors and prevent tumor recurrence, immunotherapy strategies are limited due to the low immunogenicity of tumor cells, which evade recognition by immune cells such as dendritic cells (DCs) and T lymphocytes [13]. Therefore, triggering an immune response in tumors remains a difficult challenge.

Immunogenic cell death (ICD) plays a relevant role in tumor immunotherapy and is characterized by the secretion of damage-associated molecular patterns (DAMPs), including the release of adenosine triphosphate (ATP), exposure of cell membrane surface calreticulin (CRT) and secretion of high mobility group protein B1 (HMGB1) [14, 15]. These DAMPs awaken the host immune system against tumor cells by stimulating antigen presentation of DCs and proliferation of cytotoxic T lymphocytes, which can avoid immune escape of apoptotic tumor cells due to defective molecular mechanisms. In recent years, it has been found that ferroptosis and pyroptosis can lead to ICD through the release of DAMPs [16–19]. Ferroptosis is a newly discovered form of programmed cell death that acts through the abnormal accumulation of excess iron and the loss of the cysteine-glutathione-GPX4 axis. This results in iron metabolism disorder and the accumulation of lipid peroxides, eventually causing fatal damage to cells [20]. Pyroptosis is achieved by the activation of cysteinases that mediate the formation of plasma membrane pores through members of the gasdermin protein family with high immunogenicity and the initiation of an inflammatory response, making pyroptosis a novel and potent form of ICD [21]. It has been determined that as long as some cells undergo pyroptosis, they can release tumor antigens and DAMPs, triggering an antigen-specific immune response [22]. Thus, the combined application of ferroptosis and pyroptosis has great potential in anti-tumor therapy. Both forms of cell death have been found to be caused by increases in intracellular iron and ROS levels [23, 24]. Thus, iron manipulation and the elevated ROS content may be common stimuli for both ferroptosis and pyroptosis.

Triptolide (TPL) is the main active ingredient in the Chinese herbal medicine *Tripterygium wilfordii* Hook. F., which has excellent antitumor activity [25, 26]. Studies have confirmed that TPL is more effective than other anti-tumor drugs, even drug-resistant cancers, showing excellent anti-tumor activity at nanomolar concentrations [27]. On the one hand, TPL was found to exert antitumor effects by inhibiting the expression of nuclear factor erythroid-2 related factor (Nrf2) to elevate intracellular ROS content [28]. On the other hand, in melanoma cells, the iron-mediated amplification in ROS levels could produce antitumor effects through the Tom20-Bax-caspase-GSDME pathway to induce pyroptosis [24]. Additionally, intracellularly accumulated iron can promote ferroptosis in cells. As previously mentioned, iron manipulation and ROS level elevation are common stimuli for the induction of both ferroptosis and pyroptosis, therefore, we contemplated whether TPL could induce ferroptosis and pyroptosis based on iron intervention in melanoma cells. However, the potential clinical value of TPL is severely hampered by its high toxicity, poor solubility, and limited efficiency of drug delivery to tumor sites. Recently, several strategies have been used to develop targeted TPL delivery systems, such as the use of liposomes, nanogels and polymeric micelles [29, 30]. However, these synthetic nanomaterials have potential biological toxicity in the blood and limited tumor targeting effects and are easily phagocytic and cleared [31].

Metal-organic frameworks (MOFs) are a class of materials that self-assemble through the coordination of metal ions with organic ligands. They have the advantages of mature preparation technology, large drug loading rates and good biodegradability [32, 33]. In addition, iron-containing organic nanometallic skeletons are ideal iron donors and drug delivery systems due to their porous structure to load hydrophobic drugs and large stores of iron for increasing intracellular iron. The use of bovine serum albumin (BSA) has increased in the field of drug delivery due to its biocompatibility and targeting properties, and its functional groups can be modified with folic acid (FA) to enable specific recognition of tumor cells [34–36].

**Scheme 1:**
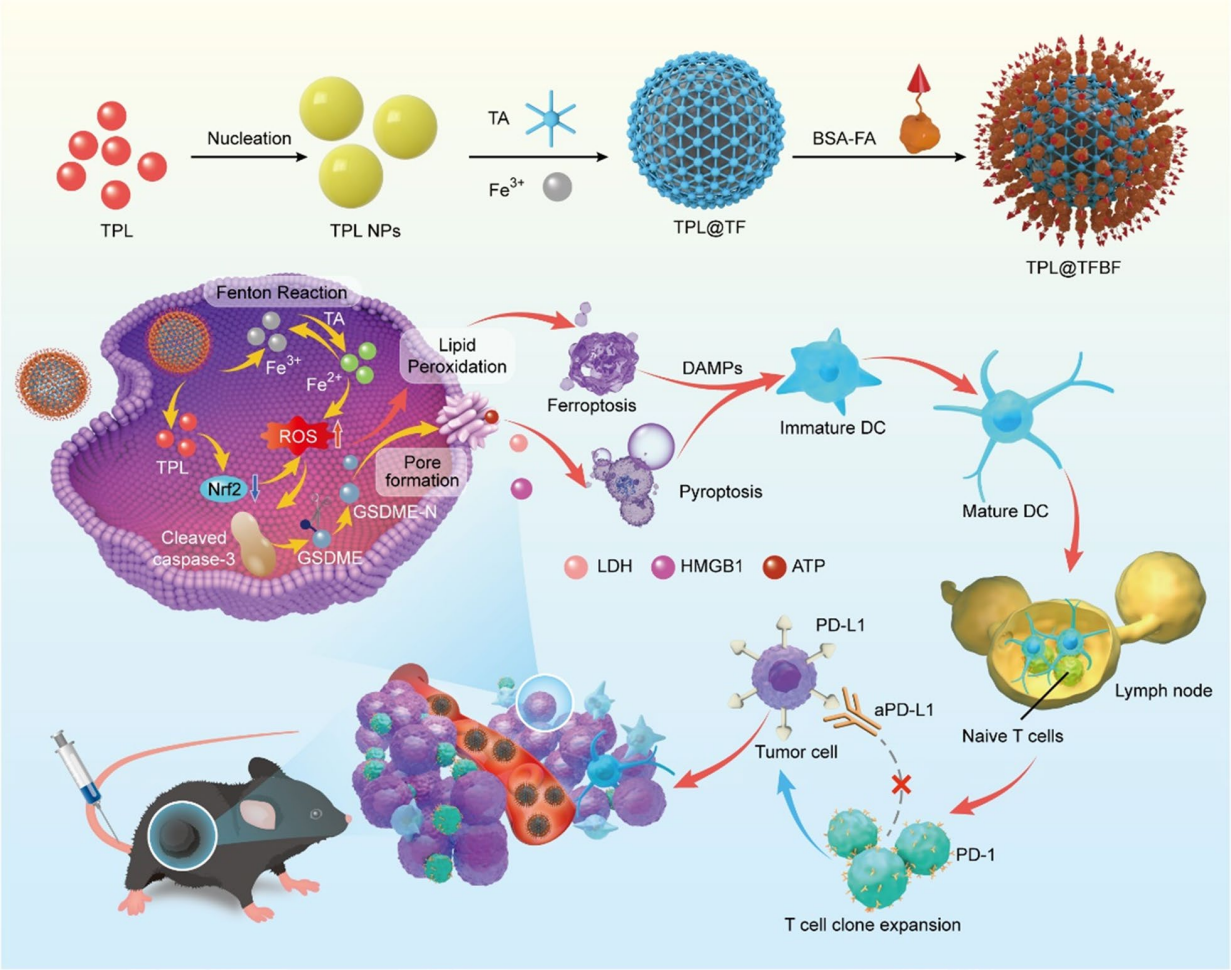
Synthesis of TPL@TFBF and its cancer immunotherapy mechanism of inducing ferroptosis and pyroptosis

Inspired by these pioneering works, we constructed an iron-containing organometalllic framework functionalized with BSA-FA (TPL@TFBF) to induce ferroptosis and pyroptosis for anti-tumor immunity (Scheme 1). Tannic acid (TA) was used as a ligand to form TA-Fe^3+^ MOFs, and the hydrophobic drug TPL was coated with BSA-FA modified MOFs. This combination of an iron-containing MOF and BSA-FA enhanced iron endocytosis and the efficiency of targeted drug delivery. After intracellular delivery, TPL@TFBF released each component to perform its respective functions: with the help of TA, Fe^3+^ was reduced to Fe^2+^ in situ, leading to ROS production through the Fenton reaction, while TPL augmented intracellular ROS production by inhibiting Nrf2 expression, both of which increase intracellular ROS amplification to induce ferroptosis and pyroptosis, resulting in the release of large amounts of DAMPs to trigger a potent systemic anti-tumor immune response to inhibit tumor growth and lung metastasis. In addition, this agent can also be combined with ICB to inhibit tumor growth through cancer immunotherapy.

## Materials and methods

### Materials, cells, and animals

#### **Materials**

Ferric chloride (FeCl_3_) and TA were obtained from Sigma Chemical Co., Ltd (Saint Louis, MO, USA). Folic acid (FA), dimethyl sulfoxide (DMSO), 1-ethyl-3-(3-dimethylaminopropyl) carbodiimide (EDC), dipotassium ethylene diamine tetraacetate (EDTA-2 K), N-hydroxysuccinimide (NHS) and triptolide were from Sinopharm Chemical Reagent Co., Ltd (Shanghai, China). Dacarbazine (DAC) was obtained from MedChemExpress (New Jersey, USA). Coomassie blue destaining solution was procured from Biosharp (Hefei, China). Bovine serum albumin (BSA) was from Aladdin Co., Ltd. (Shanghai, China). Penicillin-streptomycin solution and fetal bovine serum (FBS) were purchased from Gibco Life Technologies (Gaithersburg, MD, USA). Roswell Park Memorial Institute 1640 medium (RPMI-1640) and trypsin lysate were purchased from Procell Life Science & Technology (Wuhan, China). The Annexin V-FITC/PI double staining Apoptosis Detection Kit was obtained from BestBio (Shanghai, China). Cell Counting Kit-8 was obtained from Biosharp (Hefei, China). GSH and GSSG assay kits, reactive oxygen species assay kits, and BCA protein quantification kits were obtained from Beyotime Institute of Biotechnology (Jiangsu, China). Ferrostatin-1 (Fer-1) and Ac-DEVD-CHO (DEVD, a caspase-3 inhibitor) were from MACKLIN (Shanghai, China). Deferoxamine mesylate (DFO) was from Aladdin Co., Ltd. (Shanghai, China). Glutamic acid (Glu) and cysteine (Cys) were from BioFRoxx (Guangzhou, China). ECL chemiluminescence detection kit was obtained from Biosharp (Hefei, China). The antibodies of HMGB1, GPX4 and calreticulin polyclonal antibody were from Proteintech Group Inc. (Wuhan, China). The antibodies of GAPDH, GSDME, GSDME-N-terminal, Nrf2, CD4, CD86 and CD8 were from Affinity (China). Caspase-3 antibody was from Cell Signaling Technology (Boston, USA). Mouse tumor necrosis factor α (TNF-α) elisa kit instruction, mouse interleukin 6 (IL-6) elisa kit, mouse interleukin 1β (IL-1β) elisa kit instruction and mouse high mobility group protein B1(HMGB1) were from MEIMIAN (Wuhan, China). D-Luciferin potassium salt and Cy5.5 were purchased by MedChemExpress (New Jersey, USA). The aPD-L1 was from BioXcell (New Hampshire, USA).

#### **Cells**

Luciferase-tagged B16F10 cells (B16F10-luc cells) were kindly provided by Prof. Xiang Chen from Xiangya Hospital of Central South University. B16F10 cells were obtained from Procell Life Science & Technology (Wuhan, China). The cells were cultured with 5% CO_2_ at 37 °C using RPMI-1640 complete medium with 10% fetal bovine serum, 1% streptomycin and 1% penicillin.

#### **Animals**

C57BL/6 mice (4 to 6 weeks old) were obtained from Changzhou Cavens Laboratory Animal Co., Ltd. (Jiangsu, China). The mice were kept under SPF conditions at the Animal Laboratory Center of Hunan University of Chinese Medicine. All animal experiments adhered to ethical standards and received ethical certification (LLBH-202,211,070,004).

### ROS production and cytotoxicity in B16F10 cells after coadministration of iron and TPL

B16F10 cells (2 × 10^5^ cells per well) were inoculated in a 6-well plate. The cells were treated with PBS, Fe^3+^, TPL, and TPL + Fe^3+^ for 12 h and incubated with 2′,7′-dichlorodihydrofluorescein diacetate (DCFH-DA) probe (10 mmol/L) for 30 min. Finally, the cells were photographed by a fluorescence imaging system (NIKON, TieS, Tokyo, Japan) or quantified by flow cytometry.

B16F10 cells (5000 cells per well) were inoculated in a 96-well plate. The cells were then incubated with TPL, Fe^3+^ or TPL + Fe^3+^ for 48 h, and CCK8 solution was added for an additional 2 h of treatment. Finally, the absorbance of each well at 490 nm was measured after enzyme labelling, and the cell viability was calculated.

### Preparation of the TPL@TFBF

The TPL nanonuclear was formed by rapidly adding 100 µl of a TPL solution (10 mg/mL in DMSO) to 5 mL of ultrapure water with stirring and ultrasonication and then rapidly adding 50 µl of TA solution (40 mg/mL) and 50 µl of FeCl_3_ solution (10 mg/mL). After 2 min of ultrasound, the TPL@TF was formed. Then take the same volume of the above solution and add it to the BSA-FA solution (1 mg/ mL) and stir for 12 h. Finally, centrifuge at 16,000 rpm for 10 min to remove the supernatant and form TPL@TFBF. The concentration of TPL@TFBF was optimized by detecting the particle size and PDI of BSA (0.5, 1, 2, 4, 6 mg/mL), the particle size and PDI of TPL (100, 150, 200, 300 µg/mL), the drug loading capacity (LC%) and encapsulation efficiency (EE%).

### Quantification of iron and TPL

To determine the content of Fe in the TPL@TFBF, 5 mL of 10% nitric acid was mixed with 1 mL TPL@TFBF in a 70 °C water bath for overnight digestion. The solution was then diluted with water, filtered through a 0.45 μm polyvinylidene fluoride filter (PVDF, Millipore, USA) and finally detected by ICP-OES. To measure the TPL content, TPL@TFBF were freeze-dried and allowed to dissolve in DMSO over 3 h. The solution was centrifuged at 15,000 rpm for 10 min, and the concentration of TPL in the supernatant was determined by high-performance liquid chromatography (HPLC).

### Characterization of TPL@TFBF

A Zetasizer Nano-ZS (Malvern Instruments, UK) was used to determine the particle size and ζ potential of -the nano-MOFs. Transmission electron microscopy-energy dispersive spectroscopy (TEM-EDS, Titan G2 60–300, FEI) was used to determine the morphology and elemental composition of TPL@TFBF. The UV‒Vis absorption spectra of BSA, BSA-FA, TA-Fe, TPL@TFB, TPL and TPL@TFBF were recorded by UV-Vis spectrophotometer (UV-2600, Shimadzu, Japan). After concentrating the nanoparticles (NPs), the BSA after depolymerization of NPs was characterized by SDS-polyacrylamide gel electrophoresis.

### Stability

TPL@TFBF was incubated with PBS (10 mM, pH = 7.4), RPMI-1640 complete medium (containing 1% penicillin-streptomycin, 10% FBS), 10% FBS or H_2_O redissolved in a shaking bed at 37 °C. The particle size at predetermined time points were determined by DLS. To test their stability, TPL@TFBF was stored at 4 °C and the particle size was determined by DLS at predetermined time points.

### Cellular uptake

B16F10 cells were inoculated in 6-well plates (2 × 10^5^ cells/well), incubated with different formulations for 4 h and washed with PBS. The collected cells were resuspended in PBS, and quantitative analysis was performed by flow cytometry. In addition, cellular uptake in each group was observed using a confocal laser scanning microscope (LSM780 NLO, Zeiss, Germany). To investigate the uptake mechanism, cells were pretreated with free folic acid (FA, 1 mM) for 1 h before incubation with the nano-MOFs.

### In vitro cytotoxicity

B16F10 cells (5000 cells per well) were inoculated in a 96-well plate. Then, different concentrations of TPL, TPL@TFB or TPL@TFBF were added for 48 h of incubation, and CCK8 solution was added for an additional 2 h. Finally, the absorbance at 490 nm was measured by an enzyme marker, and cell viability was calculated.

### Fe^3+^/Fe^2+^ transformation and Fenton reaction

In order to verify whether Fe^3+^ was reduced to Fe^2+^ by TA before the application of MOFs, TFBF and TPL@TFBF were cultured with 1,10-o-phenanthroline (1 mg/mL) for 1 h, materials without the addition of 1,10-phenanthroline were used as controls. Then, and the absorbance of each reaction solution was measured at 510 nm by UV-Vis spectroscopy.

In order to verify the Fe^3+^/Fe^2+^ conversion reaction, the 1,10-o-phenanthroline colorimetric method was used to verify the content of Fe^2+^. TA (200 µg/mL), FeCl_3_ (100 µg/mL) and 1, 10-phenanthroline (1 mg/mL) were added to PBS buffers (20 mM) with pH 5.5 and 7.4. After reaction for 1 h, the absorbance of each solution was measured at 510 nm by UV-Vis spectroscopy, and Fe^2+^ production was calculated.

The Fenton reaction mediated by the nanoparticles was investigated using a methylene blue (MB) reaction. TPL@TFBF (10 µg/mL), H_2_O_2_ (1 mM) and methylene blue (MB) were added to PBS (20 mM) at pH 5.0 and 7.4, and the absorption spectra of the reaction solutions were acquired in the wavelength range of 450–720 nm using a UV-Vis spectrophotometer after 30 min of incubation.

### Intracellular ROS generation

B16F10 cells (2 × 10^5^ cells per well) were inoculated in a 6-well plate. The cells were treated with PBS, TFBF, TPL, TPL@TFB or TPL@TFBF for 12 h and then incubated with DCFH-DA (10 mmol/L) for 30 min. Finally, the cells were photographed by a fluorescence imaging system (NIKON, TieS, Tokyo, Japan) or quantified by flow cytometry.

### In vitro cell viability in the Presence of different inhibitors or promotors of Ferroptosis

B16F10 cells (5000 cells per well) were inoculated in a 96-well plate. After incubation for 24 h, TFBF, TPL, TPL@TFB or TPL@TFBF were added to each well. Fer-1 (1 µM, a ferroptosis inhibitor), DFO (100 µM, an iron inhibitor), Glu (2 mM, a ferroptosis promoter), Cys (2 mM, the precursor of cysteine) or glutathione (GSH, 2 mM, a cofactor of GPX4) were added to each well. After incubation for 24 h, the cytotoxicity was evaluated by the CCK8 method.

### In vitro cell viability in the Presence of Ac-DEVD-CHO

B16F10 cells (5000 cells per well) were inoculated in a 96-well plate. The cells were divided into TFBF, TPL, TPL@TFB and TPL@TFBF groups. Without DEVD as the control group, the cell viability of B16F10 cells after DEVD was added was detected by CCK8 method.

### Western blotting analysis (WB)

After various treatments, the cells were collected and lysed with RIPA buffer. After quantification by a BCA protein kit, the protein was separated by gel electrophoresis. The proteins were then transferred to a polyvinylidene fluoride (PVDF) membrane, which was blocked with 5% skim milk. Then, primary antibodies against GSDME, GSDME-N, caspase-3, Nrf2, CD4, CD8, CD86, HMGB1, GPX4 and GAPDH were added for incubation at 4 °C overnight, and secondary antibodies labelled with HRP were added for incubation at room temperature for 1 h. Finally, the protein was detected with a chemiluminescence imager (BioSpectrum 300, UVP).

### Cellular GSH Assay

B16F10 cells (2 × 10^5^ cells per well) were inoculated in a 6-well plate and incubated overnight for adherence. Cells were collected after 6 h of treatment with different formulations. GSH levels were determined with a GSH/GSSG test kit.

### Annexin V-FITC/PI double staining analysis

B16F10 cells were inoculated in 6-well plates (2 × 10^5^ cells per well) and treated with TFBF, TPL, TPL@TFB or TPL@TFBF for 24 h. The cells were collected and washed with PBS and then redispersed in binding solution with PI and FITC-Annexin. Finally, the cells were detected by flow cytometry.

### In vitro CRT expression

B16F10 cells (density 1 × 10^5^ cells/mL) were inoculated in a 6-well plate. After 24 h of incubation with PBS, TFBF, TPL, TPL@TFB or TPL@TFBF, the cells were collected, and anti-CRT was added. After incubation for 1 h, FITC-labelled anti-rabbit secondary antibody was added for 0.5 h of incubation, and then the cells were detected by flow cytometry.

### In vitro HMGB1 release

B16F10 cells (1 × 10^5^ cells/mL) were inoculated in a 6-well plate overnight and then treated with PBS, TFBF, TPL, TPL@TFB or TPL@TFBF for 24 h. The content of HMGB1 in the supernatant of each group was determined by ELISAs, and the level of the HMGB1 protein in the cells of each group was detected by WB.

### ATP and LDH release

B16F10 cells were inoculated in 96-well plates at a density of 5 × 10^4^ and treated with PBS, TFBF, TPL, TPL@TFB or TPL@TFBF for 24 h. The ATP and LDH released into the supernatants in different groups were detected by an ATP kit and LDH kit, respectively.

### Blood compatibility of TPL@TFBF

Hemolysis caused by the TPL@TFBF was detected, using H_2_O as a positive control and 0.9% NaCl as a negative control. Red blood cells (RBCs, 2% (v/v)) were incubated with a series of equal volumes of TPL@TFBF at 37 °C for 3 h and then centrifuged. The absorbance of the supernatant was measured at 540 nm with an enzyme label to calculate the hemolysis rate.

### In vivo imaging

B16F10 cells (2 × 10^7^ cells/ml) were injected subcutaneously into the right axilla of C57BL/6 mice to establish a mouse tumor bearing model. When the tumor size reached a certain volume, Cy5.5, TPL@TFB-Cy5.5 or TPL@TFBF-Cy5.5 were injected intravenously into B16F10 tumor-bearing mice. Images were recorded with an IVIS Lumina XRMS series instrument (PerkinElmer, Waltham, MA) 24 h after injection. Major organs and tumors were then excised from the mice and imaged ex vivo.

### In vivo antitumor efficacy

A tumor-bearing mouse model was constructed by injecting B16F10 cells (2 × 10^7^ cells/ml) subcutaneously into the right axilla of C57BL/6 mice. When the tumor volume reached ~ 80 mm^3^, the mice were randomly divided into 6 groups and injected with PBS, TFBF, TPL, TPL@TFB, TPL@TFBF or DAC (TPL: 600 ng/kg, DAC: 5 mg/kg) through the tail vein on Days 0, 3 and 6. The tumor volume was calculated every 2 days during treatment, and tumor growth curves were constructed. On Day 9, tumor tissue was obtained from the mice, photographed and weighed. Haematoxylin eosin (H&E) staining was performed on tumor tissues. The expression of the GSDME, GSDME-N, caspase-3, GPX4 and Nrf2 proteins in tumor tissues was detected by WB.

### Safety evaluation

The changes in mouse weight were recorded during treatment. H&E staining was performed on tumor tissues and major organs. Serum alanine aminotransferase (ALT), creatinine (CRE), aspartate aminotransferase (AST) and urea nitrogen (BUN) levels were detected with standard kits. Serum levels of cytokines, such as TNF-α and IL-6, were determined using the appropriate ELISA kits.

### Immune responses in tumor after treatment

After treatment, the levels of IL-6, IL-1β and TNF-α in tumor tissues were detected by mouse enzyme-linked immunosorbent assays (ELISAs). The expression of the CD86, CD4 and CD8 proteins in tumor tissues was detected by WB. Moreover, an immunofluorescence assay was used to detect the expression of immune-related proteins in tumor tissues.

### Inhibition of tumor metastasis

On Day 0, a melanoma lung metastasis model was established by intravenously injecting B16F10-luc cells into C57BL/6 mice at a density of 5 × 10^5^. The first treatment was administered on Day 1. The mice were randomly divided into 6 groups (PBS, TFBF, TPL, TPL@TFB, TPL@TFBF, or DAC) and given the appropriate drug once every two days for a total of 4 treatments, in which the dose of TPL was 600 ng/kg and the dose of DAC was 5 mg/kg. On Day 15, mice were intrabitoneally injected with D-luciferin potassium salt (150 mg/kg), lung tissue was obtained from mice and bioluminescent imaging was performed using the IVIS Lumina XRMS series (PerkinElmer, Waltham, MA).H&E staining and imaging were performed on lung sections to analyze tumor metastasis.

### Anti-tumor effects of TPL@TFBF combined with aPD-L1

In this study, B16F10 tumor-bearing mice were randomly divided into 4 groups (PBS, aPD-L1, TPL@TFBF and aPD-L1 + TPL@TFBF). The first treatment was administered on Day 0. The drugs were administered once every two days for a total of three times. aPD-L1 was administered via intraperitoneal injection at a dose of 7.5 mg/kg. All the other drugs were administered intravenously. The TPL dose was 600 ng/kg. Tumor volume was measured every 2 days. On Day 9, the tumors of the mice were excised, weighed and photographed.

### Statistical analysis

The experimental data are expressed as the mean ± SD. Two independent samples were compared using a t test, and multiple samples were compared using one-way analysis of variance (ANOVA). Significance was determined according to the following thresholds: *P < 0.05, **P < 0.01, ***P < 0.001, ****P < 0.0001.

## Results and discussion

### Effects of the combination of Fe^3+^ and TPL on ROS and cytotoxicity

**Fig. 1:**
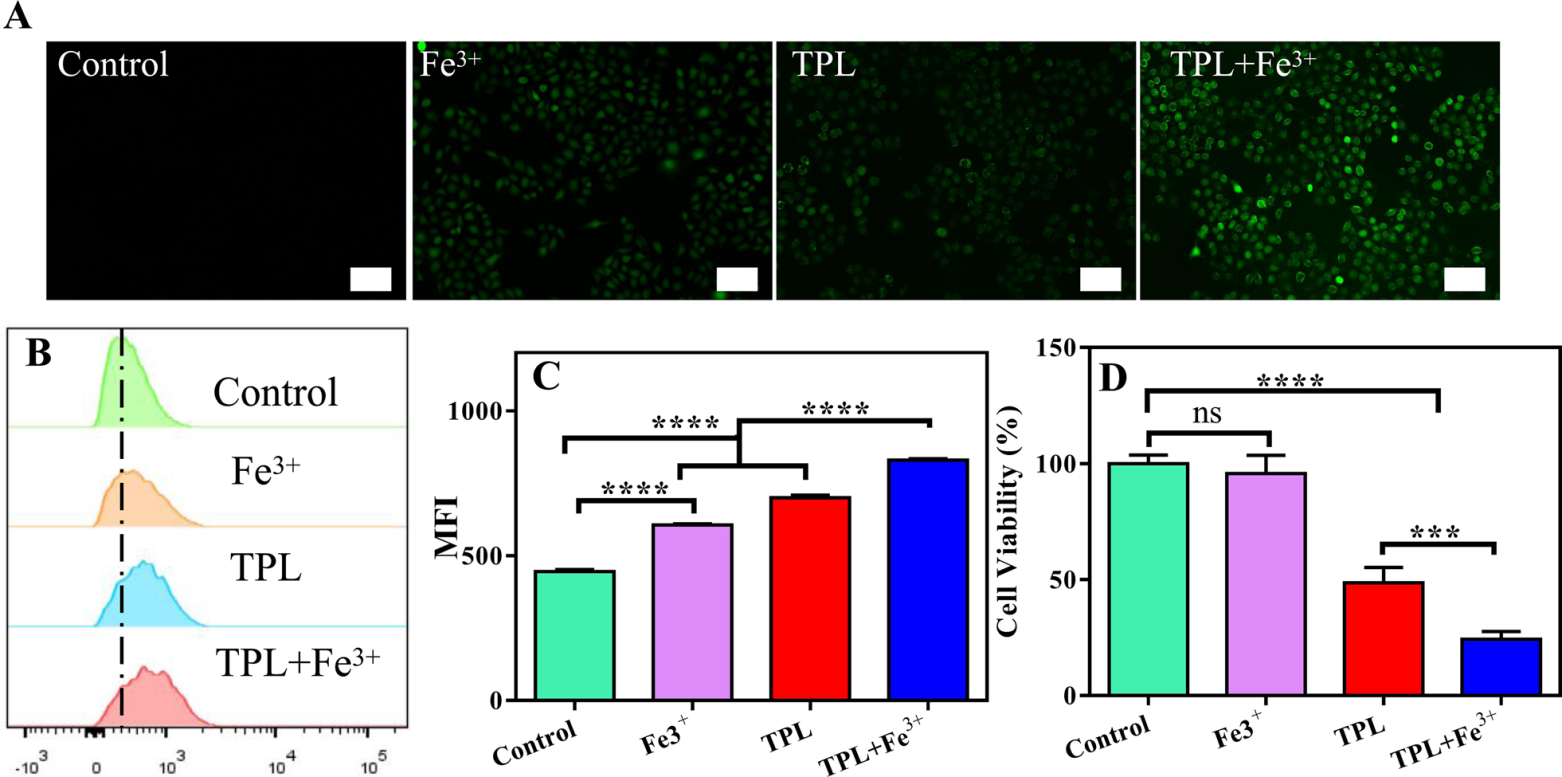
**A** Fluorescence microscopy images showing ROS levels in B16F10 cells treated with different agents. Scale bar: 50 μm. **B** Flow cytometry results of the ROS levels in B16F10 cells treated with different formulations. **C** The average fluorescence intensity of different formulations incubated with B16F10 cells. MFI: Mean fluorescence intensity. Data are expressed as the mean ± SD (n = 3). ns, not significant, ****P < 0.0001. **D** Viability of B16F10 cells incubated with different formulations for 48 h

It has been reported that TPL can inhibit the expression of Nrf2, thus inhibiting Nrf2-mediated GSH synthesis and increasing intracellular ROS levels [28]. In addition, the increase in intracellular iron can increase ROS production through the Fenton reaction [23]. Therefore, we considered whether the combination of Fe^3+^ and TPL can synergistically amplify ROS generation. First, intracellular ROS levels were measured using DCFH-DA probe. DCFH-DA itself is not fluorescent, but it can be rapidly oxidized in the presence of ROS to produce fluorescent compound 2′,7′-dichlorofluorescein (DCF), and thus the levels of ROS can be monitored dynamically by measuring the intensity of fluorescence signal. As predicted, there was weak fluorescence in the untreated group, indicating basal levels of intracellular ROS. The fluorescence intensity was enhanced in the Fe^3+^ group compared to the control group, indicating that Fe may increase the production of intracellular ROS. In addition, the fluorescence intensity of the TPL group was also enhanced compared with the control group, indicating that TPL could also induce ROS production. Surprisingly, the fluorescence intensity of the TPL plus Fe^3+^ groups was further enhanced, suggesting that Fe^3+^ could cooperate with TPL to increase ROS production after entering cells (Fig. 1A). For quantitative analysis, we measured the intracellular fluorescence intensity by flow cytometry (Fig. 1B, C), and these data were consistent with the results of fluorescence micrographs. This shows that the combination of Fe and TPL has a significant ability to synergistically amplify ROS production. Then, we initially evaluated the toxicity of Fe^3+^ in combination with TPL to B16F10 cells using CCK8 assays (Fig. 1D). We found that Fe^3+^ alone showed almost no toxicity to B16F10 cells, and although Fe^3+^ was previously shown to promote intracellular ROS levels, it was not sufficient to inhibit cell growth. Notably, Fe^3+^ in combination with TPL exhibited stronger cytotoxicity than free TPL, suggesting that TPL in combination with Fe^3+^ can synergistically amplify ROS and thus exert anti-tumor effect.

### Preparation and characterization of TPL@TFBF

**Fig. 2:**
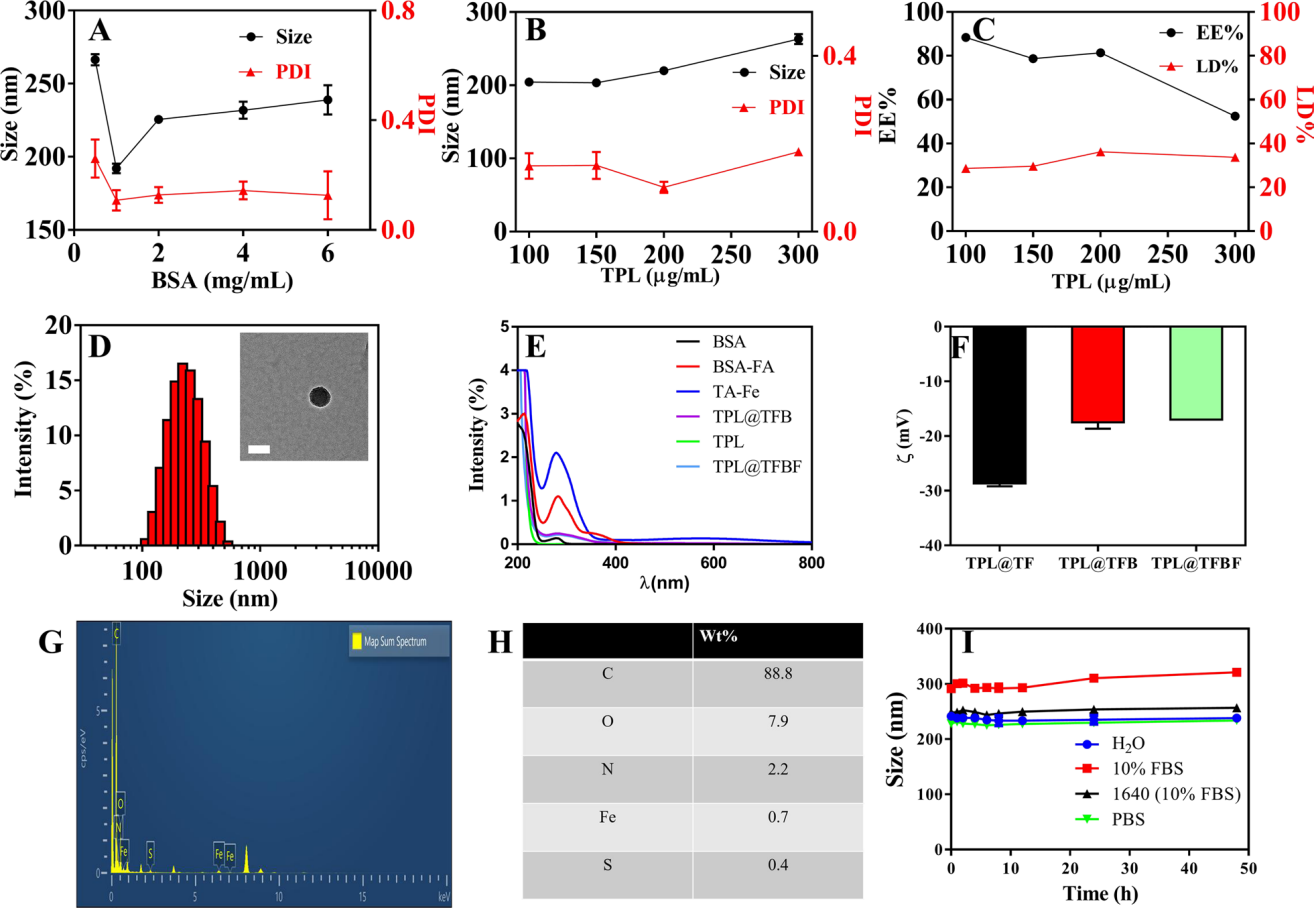
Effects of **A** BSA dosing concentration and **B** TPL dosing concentration on nanoparticle size and PDI. **C** Effect of TPL concentration on the LD% and EE% of TPL@TFBF. **D** TEM images and hydrodynamic dimensions of TPL@TFBF. Scale = 100 nm. **E** UV-vis spectra of FA, BSA, TPL, TA-Fe, BSA-FA, TPL@TFB and TPL@TFBF. **F** Zeta potentials of TPL@TF, TPL@TFB and TPL@TFBF. **G** The EDS analysis of TPL@TFBF. (H) Surface element composition of TPL@TFBF. **I** Stability of TPL@TFBF incubated in H_2_O, 10% FBS, RPMI-1640 cell medium (with 10% FBS) and PBS for 48 h

TPL is a hydrophobic drug with poor solubility in aqueous solution. Additionally, the ability of free Fe^3+^ to enter the cell is limited. Therefore, it is necessary for us to construct a nanoformulation that can supply Fe^3+^ and load hydrophobic drugs. To construct TPL@TFBF, we prepared pure drug nanonuclear via a drug self-assembly strategy, by simply dissolving the drug in DMSO solution and then adding it dropwise to an aqueous solution under ultrasonication. This was followed by the formation of a MOF shell layer by TA and Fe^3+^ coordination, and finally adding BSA to make the nanoparticles more stable. We first explored the influence of BSA dosing concentration on the particle size and polydispersity index (PDI) (Fig. 2A), and found that the particle size and PDI were minimized when the BSA dosing concentration was 1 mg/mL, therefore, we chose a BSA concentration of 1 mg/mL for subsequent preparations. Then, we optimized the drug delivery concentration of TPL, and the results showed that when the input concentration of TPL was 100 ~ 300 µg/mL, the particle size did not change significantly. However, at 200 µg/mL TPL, the PDI was the smallest and the drug loading (LD%) (36.2%) and encapsulation efficiency (EE%) (81.4%) were the highest (Fig. 2B, C); therefore, we chose a TPL input concentration of 200 µg/mL for the subsequent TPL@TFBF nanoparticle preparation.

To endow the nanostructures with tumor-targeting properties, we modified BSA with FA, a ligand for the folate receptor that is overexpressed on several types of tumor cells, including B16F10 cells [37]. BSA-FA was synthesized by our previous method [38], and its successful coupling was confirmed by UV spectroscopy (Fig. 2E). Using BSA-FA as a stabilizer, the obtained TPL@TFBF had a size of approximately 200 nm and ξ potential of approximately − 17 mV (Fig. 2D, F), both of which were very similar to those of the TPL@TFB. Therefore, the effect of coupling FA onto BSA-modified TPL nanoparticles is small. In addition, we measured the particle size and zeta potential of TPL@TF using a Malvern particle sizer (Fig. 2D, F and Additional file 1: Fig. S1) and found that the hydrodynamic particle size of TPL@TFB after BSA wrapping increased slightly from ~ 160 nm to ~ 200 nm, and the zeta potential decreased from approximately − 29 mV to approximately − 17 mV. Both of these results demonstrate that BSA was effectively modified on the surface of TPL@TF. We used SDS–polyacrylamide gel electrophoresis to characterize the BSA in the sample after TPL@TFBF depolymerization, and the data further demonstrated the successful inclusion of BSA (Additional file 1: Fig. S2). The TEM results showed that the TPL@TFBF exhibited a typical spherical morphology (Fig. 2D) with a size of approximately 100 nm. This is slightly less than that measured by DLS because DLS measures the hydrated particle size, and the expansion of BSA in aqueous solution also increases the particle size of TPL@TFBF. Elemental characterization of the TPL@TFBF was performed using EDS (Fig. 2G, H). The results showed that Fe, a characteristic element in TA-Fe(III) MOFs, and S, a characteristic element in BSA, were present in the NPs. These results showed that the BSA-modified TA-Fe(III) MOFs were successfully prepared. The nanoparticles exhibited excellent colloidal stability as well as long-term storage stability in various biological media due to the surface stabilization provided by BSA (Fig. 2I and Additional file 1: Fig. S3). Finally, the LD% and EE% of Fe in the TPL@TFBF were determined to be 3.28% and 29.52%, respectively, by ICP‒OES.

### Tumor targeting in vitro

**Fig. 3:**
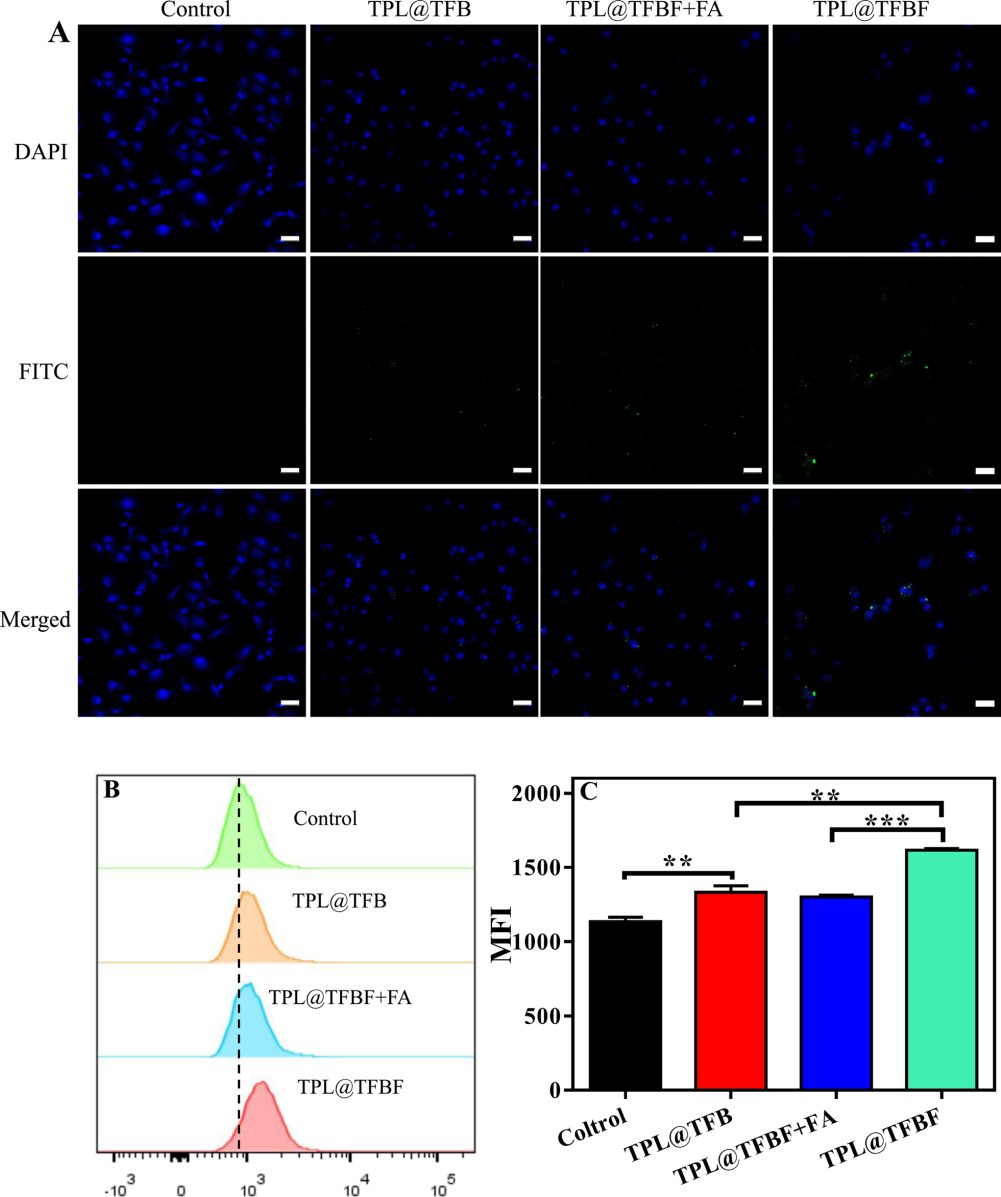
**A** CLSM images of B16F10 cells after different treatments. Scale = 50 μm. **B** Cell uptake of different formulations determined by flow cytometry analysis. **C** The quantitative results from **B**. Data are expressed as the mean ± SD (n = 3). **P < 0.01, ****P < 0.0001

Next, we studied the cellular uptake of TPL@TFBF by using B16F10 cells as a model. To track intracellular TPL@TFBF, according to our previous method [38], BSA was labelled with green fluorescein isothiocyanate (FITC), and the nucleus was colored blue with DAPI for localization. Compared with the control group, the confocal laser scanning microscopy (CLSM) images showed that cells treated with TPL@TFB showed only weak fluorescence, indicating less uptake of TPL@TFB (Fig. 3A). This may be due to the cell’s rejection of the negatively charged nanoparticles. In contrast, the intracellular fluorescence was enhanced in the TPL@TFBF group. This apparently enhanced cellular uptake may be attributed to folate receptor β (FR-β) overexpression on the membrane of B16F10 cells, promoting the cellular uptake of TPL@TFBF through a specific interaction between FA and FR. To test this, we pretreated cells with free FA to bind to and saturate the folate receptor on the membrane. As expected, this caused the intracellular fluorescence to be significantly reduced, further demonstrating that FA modification can increase the uptake of nano-MOFs. For quantitative examination, we performed flow cytometry experiments (Fig. 3B) and quantified the fluorescence intensity (Fig. 3C). Compared with the TPL@TFB group, the fluorescence intensity in the TPL@TFBF group was significantly enhanced, showing the targeting ability of these NPs. On the other hand, free FA-pretreated cells displayed elimination of this FA-mediated delivery. Overall, the flow cytometry results were in line with the CLSM results.

### In vitro analysis of TPL@TFBF

**Fig. 4:**
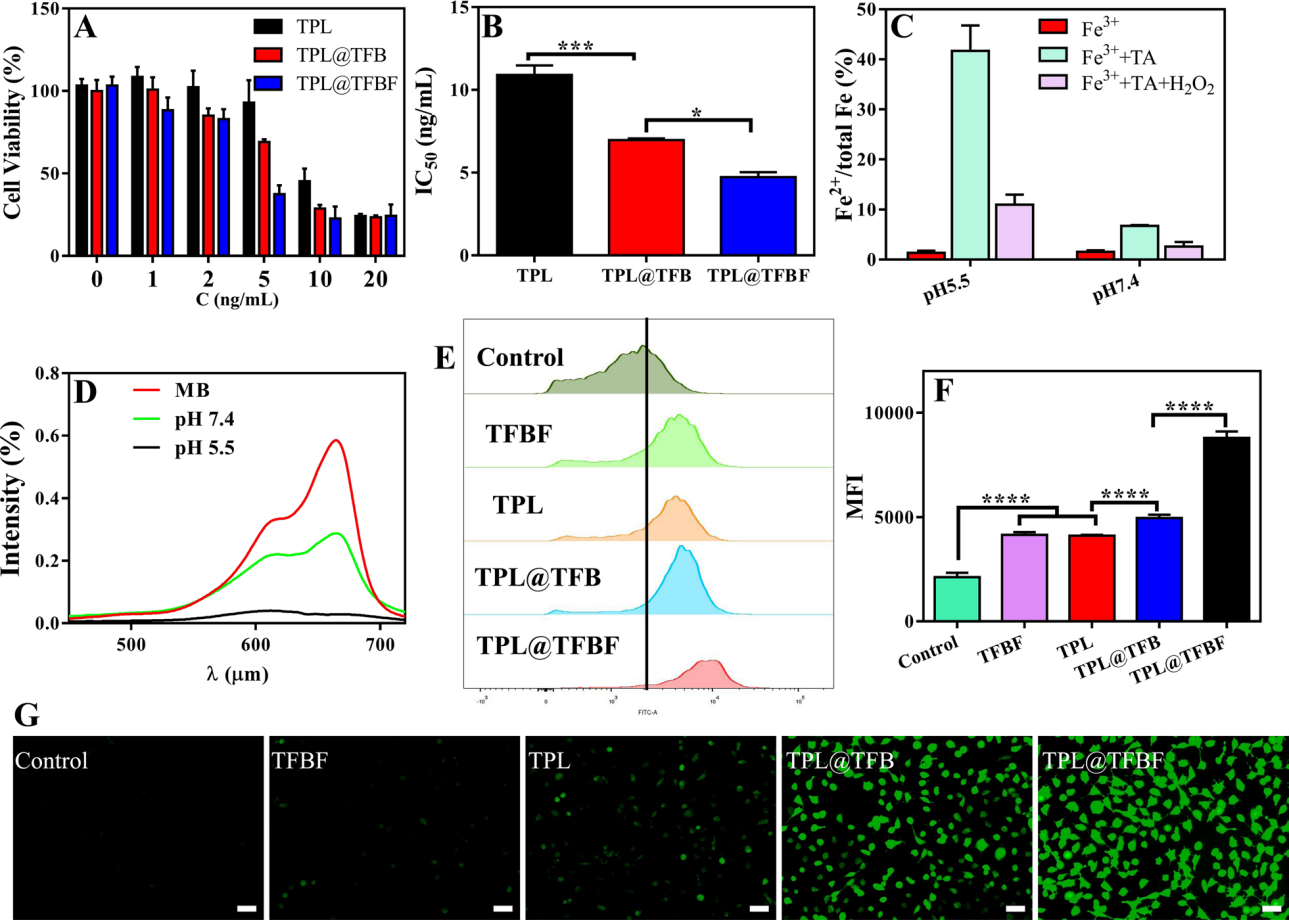
**A** Viability of B16F10 cells treated with different preparations for 48 h. C (ng/mL) indicates the concentration of TPL in each preparation. **B** IC50 values of different preparations. **C** Determination of Fe^2+^ content after different treatments. **D** Degradation of MB in the presence of H_2_O_2_ at pH 5.5 and 7.4. **E** ROS levels in B16F10 cells detected by flow cytometry after different treatments. **F** Quantification of the results from (**E**). Data are expressed as the mean ± SD (n = 4). ****P < 0.0001. **G** Fluorescence imaging of ROS in B16F10 cells treated with different preparations. Scale bar:50 μm

After demonstrating the tumor-targeted delivery, we initially evaluated the in vitro toxicity of various agents to B16F10 cells using the CCK8 assay. After 48 h of treatment with different preparations containing different drug concentrations, cell viability decreased in a dose-dependent manner (Fig. 4A), and at each concentration, the TPL@TFBF exhibited stronger cytotoxicity than free TPL and TPL@TFB, leading to significant decreases in IC50 values (Fig. 4B). This may be because the nanoparticle-mediated delivery of TPL and the cell inhibitory activity of TPL were significantly improved after it was incorporated into nanoparticles. For the TPL@TFBF, FA modification enhanced the anti-tumor effects through targeted delivery. ROS are key factors that promote lipid peroxidation and play an important role in various types of cell death, among which hydroxyl radicals play an important role in anticancer therapy [23]. The Fenton reaction mediated by iron ions can convert endogenous H_2_O_2_ into hydroxyl radicals (•OH), thereby increasing intracellular ROS levels, especially Fe^2+^, which can trigger a more efficient Fenton reaction [39]. First, we verified whether Fe^3+^ would be reduced to Fe^2+^ by performing a 1,10-O-phenanthroline colorimetric assay before TA-Fe^3+^ MOF application. As shown in Additional file 1: Fig. S4, the color in the TFBF and TPL@TFBF groups did not change significantly after the addition of phenanthroline, and the conversion of Fe^2+^ was determined to be negligible (less than 2%). This result shows that TFBF and the TPL@TFBF have a weak ability to reduce Fe^3+^ to Fe^2+^ before application. Next, we explored the ability of TPL@TFBF to generate Fe^2+^ by TA reduction of Fe^3+^ after cell entry. As shown in Fig. 4C, the conversion to Fe^2+^ was only 6.9% after the addition of TA at pH 7.4, and as the pH of the system decreased, more Fe^3+^ was reduced to Fe^2+^, reaching 40% at pH 5.5. The addition of H_2_O_2_ to the above system triggered the Fenton reaction mediated by Fe^2+^, and Fe^2+^ was converted to Fe^3+^ again. We further verified the generation of free radicals by using the fading reaction of MB. As shown in Fig. 4D, free MB has strong ultraviolet absorption at 664 nm. The addition of Fe^3+^, TA and H_2_O_2_ leads to the formation of •OH, which degrades MB and significantly reduces its ultraviolet absorption. In particular, at pH 5.5, MB was completely degraded. The above results indicated that TA could reduce Fe^3+^ to Fe^2+^ under acidic conditions, effectively triggering the Fenton reaction and converting H_2_O_2_ to •OH, confirming that the TPL@TFBF can induce the Fenton reaction in situ in the acidic tumor microenvironment. In addition, we measured intracellular ROS levels using the DCFH-DA probe. Compared with the control group, the fluorescence intensity of TFBF group was enhanced, further demonstrating that TA reduces Fe^3+^ to Fe^2+^ after TFBF enters tumor cells, triggering the Fenton reaction and increasing ROS production. The fluorescence intensity was also enhanced in the TPL group compared to the control group, implying that TPL could also induce ROS production. Unexpectedly, the fluorescence intensity of the TPL@TFB group was enhanced relative to that of the TFBF and TPL groups, implying that the Fe^3+^ entering the cells could cooperate with TPL to increase the production of ROS. In addition, the strongest fluorescence intensity was observed in the TPL@TFBF group, which may be due to the targeted delivery mediated by FA, further increasing the level of intracellular Fe^3+^ and synergistically amplifying the ROS signal (Fig. 4G). For quantitative analysis, we also measured the intracellular fluorescence intensity by flow cytometry (Fig. 4E, F). The fluorescence intensity of the TPL@TFBF group was 4 times higher than that of the untreated group and significantly different compared with the other groups, which was in accordance with the photographs. This shows that the TPL@TFBF can significantly and synergistically amplify ROS production through the binding of Fe^3+^ and TPL.

### Verification of anti-tumor mechanism of action

In summary, there is a synergistic amplification of ROS production by the combination of Fe^3+^ and TPL, which led to a significant enhancement in the efficacy of the TPL@TFBF. We next examined the potential mechanisms. As we all know, Nrf2 has long been considered an important component of the antioxidant system that regulates the expression of antioxidant genes, thereby counteracting oxidative and pro-electrical stress [40]. In addition, a series of redox-related genes targeted by Nrf2 are key mediators of ferroptosis. For example, the inhibition of GPX4-induced ferroptosis could be reversed by overexpressing Nrf2 [41]. However, TPL can directly inhibit the expression of Nrf2 and interfere with de novo synthesis of glutathione [28], thereby increasing ferroptosis sensitivity and producing antitumor effects. On the other hand, the Fe^3+^ mediated Fenton reaction can elevate intracellular ROS levels to induce ferroptosis. In addition, manipulating the content of intracellular iron was found to induce pyroptosis [23]. Therefore, the combination of TPL and Fe^3+^ may enhance the therapeutic effect by synergistically amplifying ROS production, thereby causing simultaneous ferroptosis and pyroptosis. We next examined whether TPL@TFBF could induce both ferroptosis and pyroptosis.

**Fig. 5:**
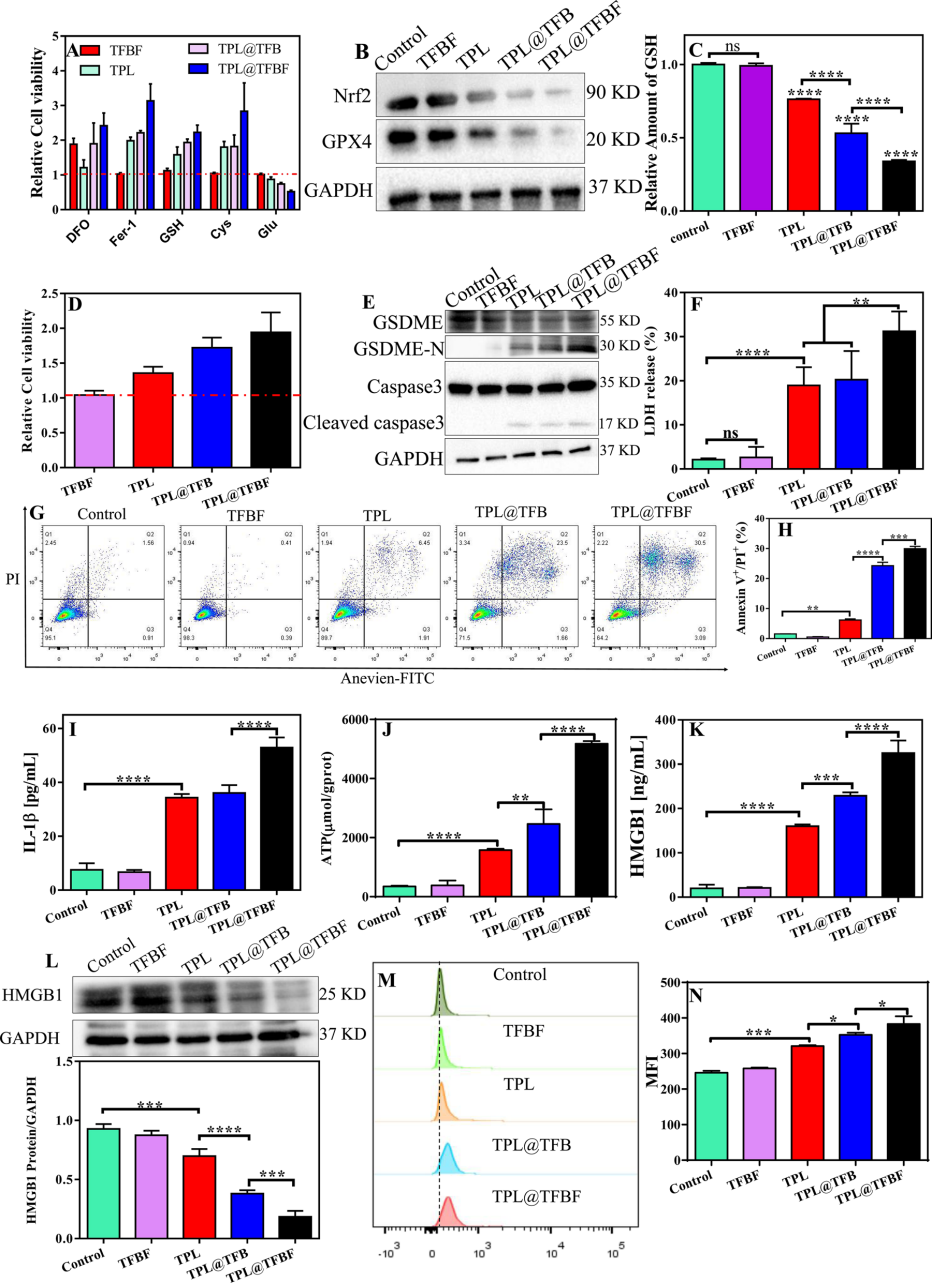
**A** Relative cell viability of B16F10 cells after incubation in different preparation groups (TFBF, TPL, TPL@TFB and TPL@TFBF with and without various compounds). **B** Expression of Nrf2 and GPX4 proteins after different treatments. **C** Relative GSH levels in B16F10 cells after different preparations. Data are expressed as the mean ± SD (n = 3). ns, not significant, ****P < 0.0001. **D** Relative cell viability (compared to the group without inhibitor treatment) of B16F10 cells after different treatments in the presence of DEVD. **E** Expression of GSDME, GSDME-N and cleaved-caspase-3 proteins after different treatments. **F** LDH release from B16F10 cells after different treatments. Data are expressed as the mean ± SD (n = 3). ns, not significant, **P < 0.01, ****P < 0.0001. **G** Annexin V/PI double staining analysis of B16F10 cells treated with different preparations. **H** Annexin V^+^/PI^+^ quantification of (**G**). Data are expressed as the mean ± SD (n = 3). **P < 0.01, ***P < 0.001, ****P < 0.0001. Release of IL-1β (**I**), ATP (**J**) and (**K**) HMGB1 from B16F10 cells after treatment with different preparations. **L** Expression of the HMGB1 protein in B16F10 cells after different treatments. Data are presented as the mean ± SD (n = 3). ***P < 0.001, ****P < 0.0001. **M** Flow cytometric results of CRT exposure from B16F10 cells treated with different agents and (**N**) quantitative average fluorescence intensity. Data are expressed as the mean ± SD (n = 3). *P < 0.05, ***P < 0.001

First, B16F10 cells were treated with various ferroptosis inhibitors/promoters in combination with various preparations, and the relative cytotoxicity was calculated by comparison with the group not treated with ferroptosis inhibitors/promoters. As shown in Fig. 5A, the ferroptosis inhibitor Fer-1 alleviated the cytotoxicity of the TPL@TFBF. Similarly, the iron chelator DFO, the ferroptosis inhibitor Cys, and the ferroptosis detoxifier GSH attenuated the cytotoxicity of TPL@TFBF. In contrast, Glu, as a ferroptosis inducer, achieved the opposite effect by inhibiting Cys uptake. Overall, all these results demonstrate that the constructed of TPL@TFBF can effectively promote ferroptosis. To further explore the mechanism by which TPL@TFBF induces ferroptosis, the expression levels of Nrf2 and GPX4 were detected by WB. GPX4 is a marker of ferroptosis. Compared with that in the control and TFBF groups, the expression of Nrf2 and GPX4 was downregulated in B16F10 cells treated with TPL, TPL@TFB and TPL@TFBF, and notably, their expression was the lowest in the TPL@TFBF group (Fig. 5B), suggesting that the codelivery of TPL and Fe^3+^ could significantly reduce the expression of Nrf2 and GPX4. Quantification of the protein levels further confirmed this significant change (Additional file 1: Fig. S5). Correspondingly, intracellular GSH levels showed the same downwards trend (Fig. 5C). Since GPX4 requires the assistance of GSH to participate in lipid peroxidation, the downregulation of GSH induced by TPL can promote the downregulation of GPX4 to induce ferroptosis. By verifying these two key factors required for ferroptosis (GSH reduction and GPX4 downregulation), it was confirmed that the TPL@TFBF could induce ferroptosis.

TPL has been found to induce GSDME-dependent pyroptosis [42], and iron-activated ROS have previously been reported to induce pyroptosis via the Tom20-Bax-caspase-GSDME pathway [24]. Therefore, further research was needed to determine whether GSDME-dependent pyroptosis also contributes to TPL@TFBF-induced cell death. GSDME-dependent pyroptosis is mainly activated by caspase-3. After activation, the GSDME protein hydrolyses and releases N fragments, which assemble to form pores on the cell membrane. The cell swelling and plasma membrane rupture result in the destruction of membrane integrity and the release of cellular contents [43, 44]. To determine whether TPL@TFBF induced pyroptosis via the GSDME pathway, we treated B16F10 cells with DEVD. As shown in Fig. 5D, the cytotoxicities of TPL, TPL@TFB and TPL@TFBF were significantly reduced by the addition of DEVD, and the TPL@TFBF showed a stronger effect, suggesting that TPL@TFBF-induced cell death may depend on the caspase-3-mediated pathway. To further explore the pyroptosis mechanism of TPL@TFBF treatment of B16F10 cells, we examined the levels of pyroptosis-associated protein in B16F10 cells using WB. As shown in Fig. 5E, cleaved caspase-3 and GSDME-N expression were increased in the TPL, TPL@TFB, and TPL@TFBF groups, and GSDME-N was more strongly upregulated in the TPL@TFBF group than in the other groups. These results indicated that free TPL could induce pyroptosis mediated by caspase-3, and the addition of Fe^3+^ could better induce pyroptosis. This phenomenon was further confirmed by semiquantitative analysis (Additional file 1: Fig. S6), which showed that the expression level of GSDME-N in the TPL@TFB group was 1.5 times that in the TPL group and that in the TPL@TFBF group was 1.5 times that in the TPL@TFB group. These results suggest that TPL@TFBF can activate caspase-3 to cleave GSDME into GSDME-N and further induce pyroptosis. Subsequently, we evaluated the release of lactate dehydrogenase (LDH, an indicator of pyroptosis) and IL-1β into the cell supernatant to examine the leakage of intracellular contents. The release of LDH and IL-1β into the supernatant of TPL@TFBF-treated cells was significantly higher than that in the other treatment groups (Fig. 5F and I). To further demonstrate the ability of the TPL@TFBF to induce pyroptosis, we used Annexin V-FITC/PI double staining analysis as reported in the literature [45–47]. As shown in Fig. 5G, H and Additional file 1: Fig. S7, B16F10 cells underwent significant pyroptosis after TPL@TFBF treatment. Moreover, there were very few early apoptotic cells, which can rule out the occurrence of apoptosis. Thus, TPL@TFBF enhanced caspase-3 mediated GSDME cleavage and increased the release of IL-1β and LDH, confirming that TPL@TFBF is a good inducer of pyroptosis.

### Induction of ICD

Previous studies have shown that both pyroptosis and ferroptosis may lead to immunogenic cell death (ICD), so there is an opportunity to use this combination with immunotherapy for synergistic effects [48–50]. To explore this effect, we next studied the DAMPs released from dying cells, including HMGB1, ATP and CRT, which are important features of ICD. We first examined the release of ATP and HMGB1 into the cell supernatants, and extracellular ATP and HMGB1 had been released in the TPL, TPL@TFB and TPL@TFBF groups in comparison with the control and TFBF groups, especially in TPL@TFBF groups (Fig. 5J, K). We also detected intracellular HMGB1 levels by WB. Compared with the control and TFBF groups, TPL, TPL@TFB and TPL@TFBF were all effective in reducing HMGB1 concentration, and the reduction effect of TPL@TFBF was more significant (Fig. 5L). We then examined CRT exposure by flow cytometry, and the results showed that TPL@TFBF significantly increased CRT exposure (Fig. 5M); moreover, quantitative analysis showed that the CRT exposure induced by the TPL@TFBF was significant compared with that in the other treatment groups (Fig. 5N). These results suggest that the ability of TPL@TFBF to induce immunogenicity is enhanced to some extent by FA modification due to tumor targeting activity. Overall, cells experienced ICDs after treatment with TPL@TFBF, which could be attributed to pyroptosis and ferroptosis cytotoxicity induced by nano-MOFs.

### In vivo performance of nano-MOFs

**Fig. 6:**
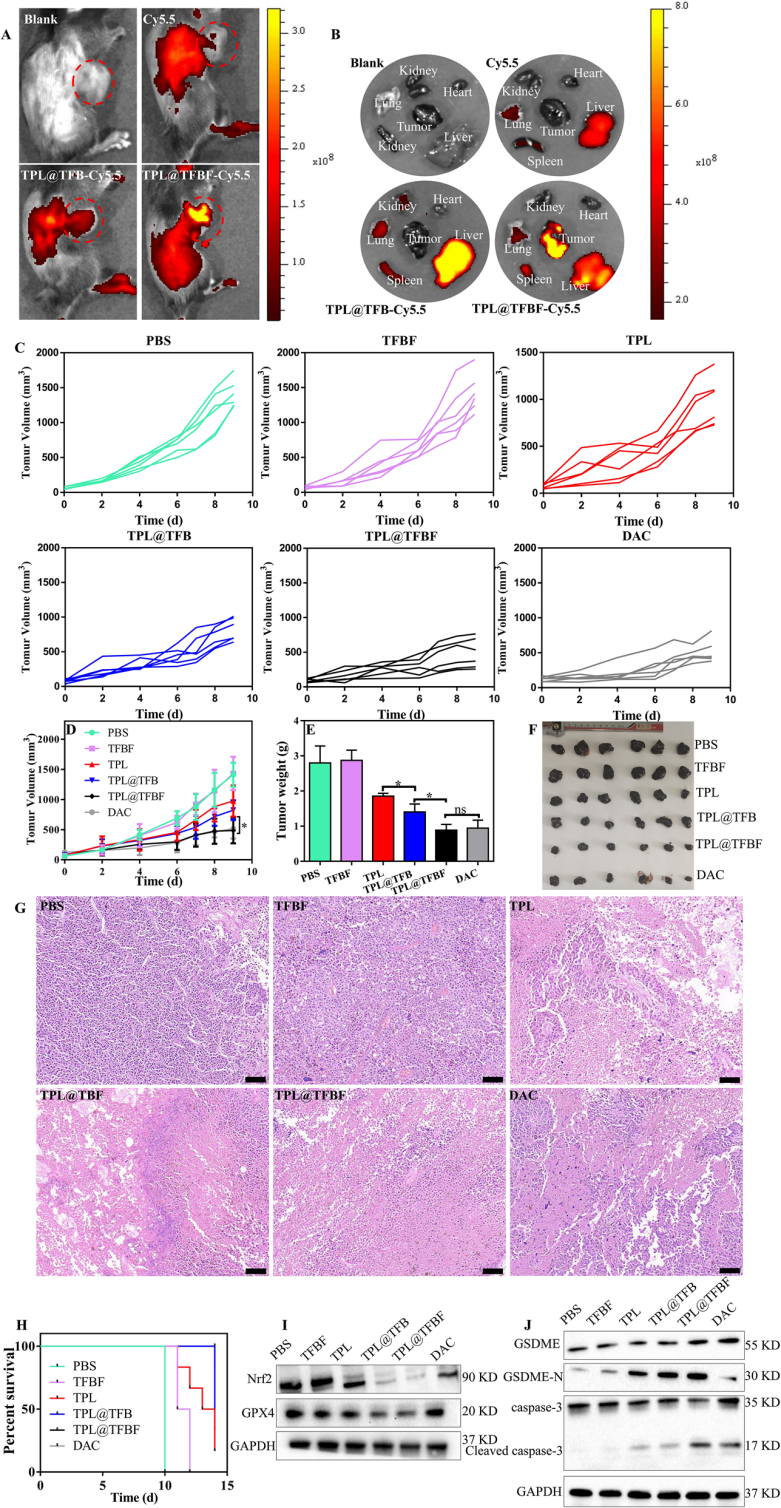
Fluorescence images of tumor-bearing mice in vivo **A** and in vitro **B** after different treatments. **C–D** The tumor growth curves of mice post different treatments. **E** Weight, **F** histograms, and **G** H&E staining of tumors in each group at the end of treatment. Scale bar, 100 μm. Data are expressed as the mean ± SD (n = 6). ns, not significant, *P < 0.05. **H** Survival rates of mice treated with each preparation. **I** The expression levels of Nrf2 and GPX4 in different groups of tumor tissues were detected by WB. **J** Expression levels of GSDME, GSDME-N, cleaved caspase-3 in tumor tissues after treatment

As we demonstrated, TPL@TFBF can induce ferroptosis and pyroptosis in vitro. Therefore, we hypothesized that TPL@TFBF can also inhibit tumor growth in vivo. Next, we applied a B16F10 tumor-bearing mouse model to characterize the performance of TPL@TFBF in vivo. Blood compatibility tests were performed before the nanoparticles were injected intravenously. At concentrations as high as 200 µg/ml, hemolysis rates of less than 2% were observed after with TPL@TFBF (Additional file 1: Fig. S8), indicating their high biosafety after intravenous administration. To study the in vivo biodistribution, TPL@TFBF was labelled with Cy5.5. After 24 h of nanoparticle injection, the fluorescence of the nanoparticles was much stronger than that of free Cy5.5, which may be attributed to the longer circulation half-life of the nanosystem. It is worth noting that significant fluorescence from the nanoparticles was observed in tumor tissue, indicating tumor accumulation through the EPR effect, especially for the FA-modified nanoparticles, indicating the active targeting capability of the nanoparticles in vivo (Fig. 6A). In addition, we extracted the major organs and tumor tissues of the mice for ex vivo fluorescence imaging (Fig. 6B) and observed significant fluorescence from TPL@TFBF-Cy5.5 in the tumor tissues, which was consistent with the in vivo results. Overall, our nano-MOFs can actively target tumors to deliver drugs, which will be beneficial for improving therapeutic efficacy and minimizing nonspecific side effects.

In order to evaluate the anti-tumor effects in vivo, tumor-bearing mice were randomly divided into 6 groups and treated with different formulations. The tumor size was measured to monitor the dynamic efficacy. The tumor volume increased rapidly during treatment in the PBS and TFBF groups, indicating that TFBF had no tumor suppressive effect. Compared with the PBS group, the free TPL group had moderate tumor growth inhibition (~ 30.97%), and the TPL@TFB group showed better tumor control than TPL (~ 41.74%), suggesting a synergistic effect from TPL and Fe^3+^ in vivo. The tumor suppression effect of TPL@TFBF (~ 65.58%) was enhanced to some extent by FA modification, almost reaching that of the positive drug DAC (~ 63.47%), probably due to tumor targeting (Fig. 6C,  D). Tumor tissues were extracted after treatment for direct comparison (Fig. 6E, F), and the tumor weights and corresponding tumor images were consistent with the overall trend of tumor volume. We further examined the tumor tissue by H&E staining to evaluate the therapeutic effect. In Fig. 6G, we observed significant cell necrosis and nucleopaenia in the TPL@TFBF and DAC groups. In addition, we constructed survival curves for each group (Fig. 6H), and the survival rate was 16.67% in the free TPL group and 83% in the TPL@TFBF group after 14 days, indicating that the TPL@TFBF significantly prolonged the survival of mice compared with free TPL. Thus, the above results suggest that TPL@TFBF can improve the efficiency of melanoma treatment and alleviate side effects.

To further investigate the tumor suppression mechanism of action, we assessed the expression levels of GPX4, Nrf2, cleaved caspase-3 and GSDME-N in the tumor region (Fig. 6I, J). In line with the results of the in vitro analysis, GPX4 and Nrf2 at the TPL@TFBF-treated mouse tumor sites were significantly downregulated, and cleaved caspase-3 and GSDME-N were significantly upregulated compared with the TPL and TPL@TFB groups, indicating that TPL@TFBF could exert anti-tumor effects by inhibiting Nrf2 expression and elevating intracellular ROS, thereby inducing ferroptosis and pyroptosis.

Moreover, the safety of the nanosystem was evaluated. There was little change in the weights of the mice during treatment (Additional file 1: Fig. S9), indicating no acute toxicity. Analysis of the serum biochemical indices after treatment showed that liver function and renal function were within the normal range (Additional file 1: Fig. S10), indicating that the nano-MOFs had no hepatotoxicity or renal toxicity. Further H&E staining analysis of major organs showed no pathological changes (Additional file 1: Fig. S11). In addition, to determine whether the release of various inflammatory factors from ferroptosis and pyroptosis could cause cytokine storm syndrome, we also measured the changes in serum TNF-α and IL-6 levels, and the changes after various treatments were negligible (Additional file 1: Fig. S12). Overall, our nano-MOF is a platform with high biosafety.

### Antitumor immunity induced by TPL@TFBF

**Fig. 7:**
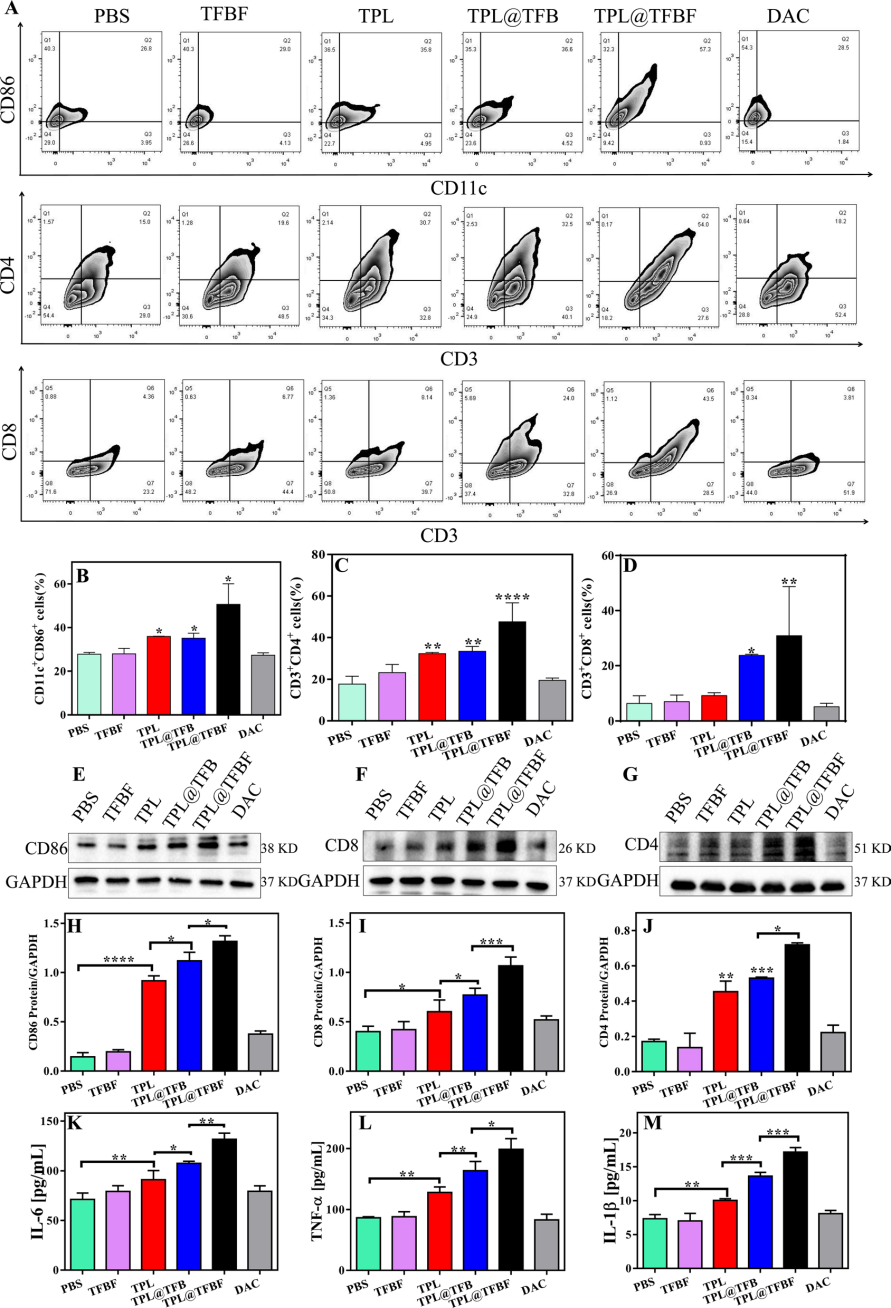
**A** Flow cytometry analysis of CD11c^+^CD86^+^ cells, CD3^+^CD4^+^ cells and CD3^+^CD8^+^ cells in tumor tissues from mice in each group after treatment with different formulations. **B–D** Quantification of the results from (**A**). **E–G** Expression levels of CD86, CD8 and CD4 in tumor tissues from different groups. **H**–**J** Quantification of the results from (**E–G**). The levels of **K** IL-6, **L** TNF-α and **M** IL-1β in tumor tissues from mice in different groups after different treatments. Data are expressed as the mean ± SD (n = 3). *P < 0.05, **P < 0.01, ***P < 0.001, ****P < 0.0001

To study the in vivo immune effect, we removed tumors after euthanasia of the mice and detected the proportion of immune cells in the tumor tissue by flow cytometry. First, mature dendritic cells (mDCs) and tumor-infiltrating T cells were detected by flow cytometry. The results showed that compared with PBS or TFBF, the proportion of activated DCs (CD86^+^CD11c^+^) in TPL@TFBF tumor tissues increased from 36.6 to 68.85%, indicating an increased level of DCs maturation after TPL@TFBF treatment (Fig. 7A, B and Additional file 1: Fig. S13). This was due to the simultaneous occurrence of ferroptosis and pyroptosis after treatment with the TPL@TFBF, which leads to a stronger immune effect, thus amplifying the maturation of DCs. After DCs maturation, antigens are presented to T cells to initiate T-cell cloning and proliferation. We monitored the ratio of cytotoxic T cells (CD8^+^) and helper T cells (CD4^+^) (Fig. 7A, C and D) and found that tumor infiltration of cytotoxic T lymphocytes (ctls, CD3^+^CD8^+^ T cells) and helper T cells (Ths, CD3^+^CD4^+^ T cells) the TPL@TFBF group was significantly higher than that in other groups. These results suggest that TPL@TFBF treatment can promote T-cell clonal expansion. Then, the expression levels of immune-related proteins in mouse tumor tissues were investigated by WB (Fig. 7E, J). After TPL treatment, the expressions of CD86, CD4 and CD8 in mouse tumor tissues were observably up-regulated. The expression of the above proteins was further up-regulated after treatment with TPL@TFB, and again after treatment with TPL@TFBF, indicating the synergistic immune activation effect of TPL and Fe^3+^. The expression of the above proteins was further investigated by immunofluorescence, and the results were basically consistent with those of WB, indicating that TPL@TFBF treatment initiated an adaptive immune response (Additional file 1: Fig. S14). T cells are activated and secrete cytokines such as tumor necrosis factor α (TNF-α) and interleukin 6 (IL-6), which directly kill tumor cells. The levels of TNF-α and IL-10 in mouse tumor tissues were significantly increased after treatment with TPL@TFBF (Fig. 7K and L), which also indicated the effective activation of anti-tumor immunity. In addition, IL-1β levels of pyroptosis associated inflammasomes were elevated, suggesting pyroptosis (Fig. 7M). Together, these in vivo experimental results show that TPL@TFBF can cause ferroptosis and pyroptosis in cancer cells, enhance immunogenicity, promote DCs activation, and trigger a T-cell-dependent immune response for excellent anti-tumor efficacy and efficient stimulation of adaptive anti-tumor immunity.

### TPL@TFBF inhibits tumor metastasis, and its combination with aPD-L1 enhances immunotherapy

**Fig. 8:**
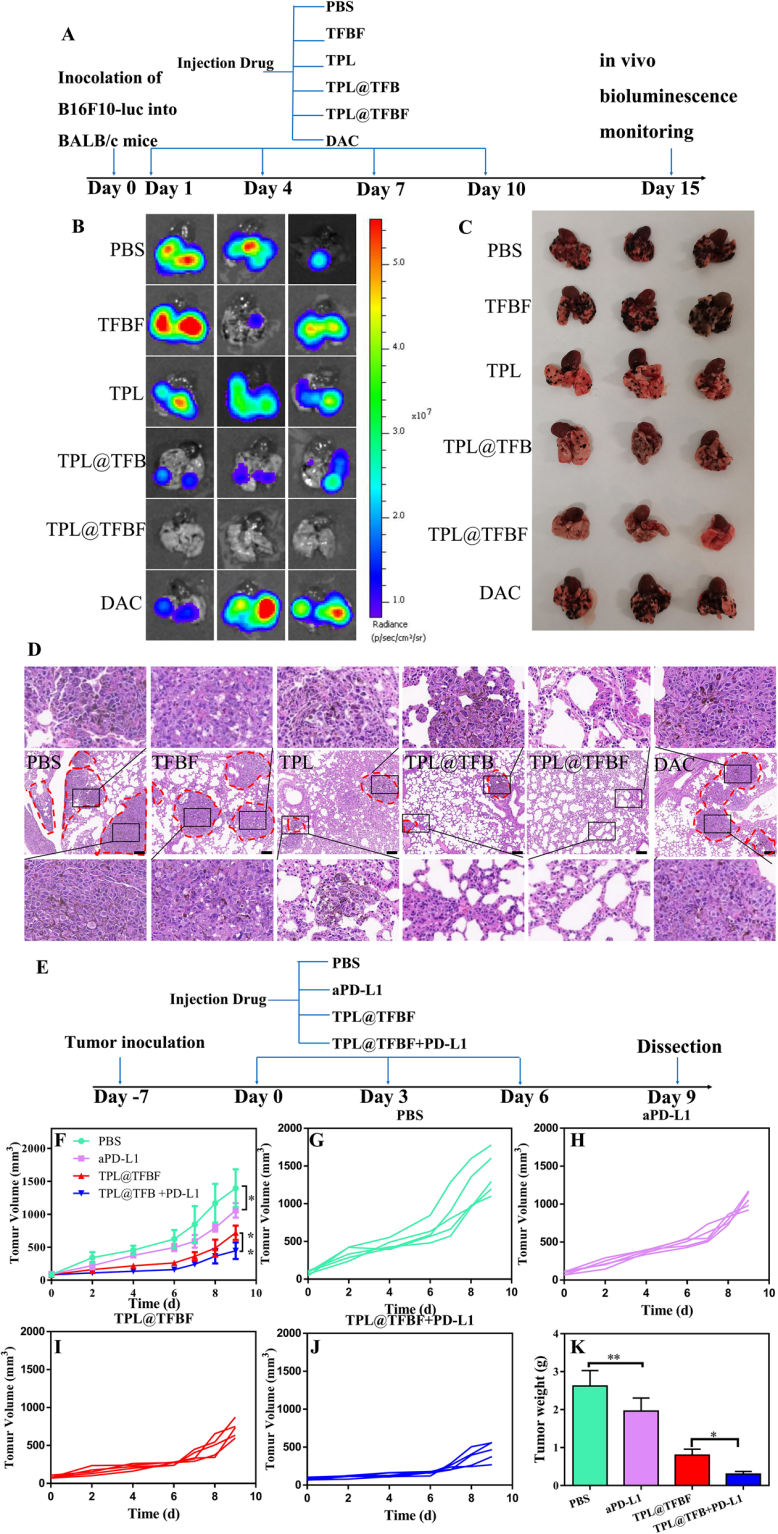
**A** Schematic diagram of the different B16F10-luc lung metastatic tumor treatments. **B** Bioluminescence imaging, **C** direct view and **D** H&E staining of lung tissue from mice with metastases after intervention with different preparations. **E** Schematic diagram of the dosing regimen for combination aPD-L1 treatment. **F–J** Tumor growth curves during different treatments. **K** Tumor weights after different treatments. Data are expressed as the mean ± SD (n = 5). *P < 0.05, **P < 0.01

The main cause of death in patients with clinical melanoma is due to their susceptibility to lung metastasis, which is also a major challenge in cancer treatment. Fortunately, TPL has been shown to inhibit the transfer of B16F10 cells to the lungs and spleens of mice. In addition, immunotherapy has the potential to inhibit metastasis. As indicated above, our nano-MOFs produce a good immune response. We constructed a lung metastatic tumor model by intravenously injecting B16F10-luc cells and evaluated the ability of TPL@TFBF to inhibit tumor metastasis. After different treatments, in vitro bioluminescence imaging was performed using fluorescein as the substrate (Fig. 8A). The lung tissue from the PBS group showed a strong fluorescence signal, indicating the successful construction of the mouse lung metastasis model. There was no significant change in the fluorescence signal in the TFBF and DAC groups, and the effects of TFBF and DAC on lung metastasis were apparently limited. After treatment with TPL and TPL@TFB, the fluorescence signal in mouse lung tissue were significantly reduced, indicating their inhibition of lung metastasis. After treatment with TPL@TFBF, there was almost no fluorescence signal in the lung tissue, indicating that metastasis activation was completely suppressed, which could be attributed to the strong immune activation produced by the TPL@TFBF (Fig. 8B).

Next, lung tissues were collected for metastatic site analysis. A large number of pulmonary metastatic nodules with melanoma cell aggregates were observed in the PBS group, while no obvious metastatic nodules were observed in the TPL@TFBF group, confirming that TPL@TFBF can effectively inhibit pulmonary metastasis (Fig. 8C). To confirm this, we performed further H&E staining evaluations on each type of lung tissue, and the lung metastatic are circled with red dashed circles in Fig. 8D. Large tumor metastases were visible in the PBS group, TFBF group and DAC group, only a few smaller metastases remained in the TPL group and TPL@TFB group, and no obvious metastases were seen in the TPL@TFBF group, confirming that TPL itself could inhibit lung metastasis, but TPL@TFBF might further inhibit lung metastasis due to activation of the immune response.

We further used the nanosystem in combination with the ICB PD-L1 antibody (aPD-L1) for melanoma for tumor co-immunotherapy. Establishment of the mouse model and the comprehensive treatment protocol are shown in Fig. 8E. Notably, the single agent aPD-L1 showed only marginal tumor suppression. TPL@TFBF was superior to aPD-L1 due to its strong immunomodulatory effect, which were markedly enhanced when used in combination with aPD-L1 (Fig. 8F, K and Additional file 1: Fig. S15). In this combination, TPL@TFBF promotes DCs activation and T-cell infiltration, while aPD-L1 activates T cells to attack tumor cells, producing a synergistic anti-tumor effect.

## **Conclusion**

In conclusion, we developed a dual-modal TPL@TFBF nanoplatform that can release DAMPs by inducing cells to undergo ferroptosis and pyroptosis and activate systemic anti-tumor immune responses to eliminate solid tumors. This nanoplatform has good drug loading efficiency and stability. In addition, the nanoplatform targets tumors due to the FA modification. First, the level of intracellular Fe^3^^+^ is significantly elevated in response to iron-containing MOF entry into cells via FA-mediated endocytosis, and the Fe^3+^-mediated Fenton effect can elevate intracellular ROS levels. Second, TPL synergistically elevates the content of cytotoxic ROS by inhibiting Nrf2 expression. On the one hand, the nanoplatform induced ferroptosis by inactivating GPX4, and on the other hand, it induced pyroptosis by activating caspase-3 and GSDME. The DAMPs released after ferroptosis and pyroptosis can promote DCs maturation, initiate T-cell clonal expansion, activate anti-tumor immunity, effectively inhibit primary tumor growth and metastasis, and prolong mouse survival time. In addition, the anti-tumor effects were further enhanced by combination with aPD-L1. Therefore, the dual induction of ferroptosis and pyroptosis by this nanoplatform has great promise for immunotherapy of solid tumors.

### Supplementary Information

**Additional file 1: Figure S1.** The particle sizes of TPL@TF and TPL@TFB. **Figure S2.** SDS-PAGE gel images of BSA and TPL@TFBF. **Figure S3.** TPL@TFBF particle size and PDI at 4℃ for 30 days. **Figure S4.** Determination of Fe^2+^ content under different treatments. **Figure S5.** Quantified expression of (**A**) Nrf2 and (**B**) GPX4. Data are expressed as the mean ± SD (n = 3). ns, not significant, *P<0.05. **Figure S6.** Quantified expression of (**A**) Cleaved caspase-3 and (**B**) GSDME-N. Data are expressed as the mean ± SD (n = 3). *P<0.05, ***P<0.001. **Figure S7.** The process of gating for pyroptosis cells in these cells in flow cytometry analysis. **Figure S8.** Hemolysis percentage of red blood cells at various concentrations of TPL@TFBF. Water treated cells were used as positive control. The negative control was 0.9% NaCl. **Figure S9.** Dynamically monitoring the body weight change during treatments (n = 6). **Figure S10. **Evaluation of the hepatotoxicity (**A**) and nephrotoxicity (**B**) of each formulation by measuring the serum levels of ALT, AST, BUN and CRE after treatments. **Figure S11.** H&E staining of heart, liver, spleen, lung and kidney after different treatments. Scale bar: 100 μm. **Figure S12.** The levels of (**A**) IL-6 and (**B**) TNF-α in serum of mice in different groups after different treatments. **Figure S13.** The process of gating for (**A**) activated DCs and (**B**) CD4+ and CD8+ T cells in these cells in flow cytometry analysis. **Figure S14.** (**A**) The expression of CD4 and CD8 proteins in tumor tissue after different treatments. (**B**) The expression of CD11c and CD80 proteins in tumor tissue after different treatments. Scale bar: 20 μm. **Figure S15.** Representative photographs of resected tumors.

## Data Availability

The raw data and processed data required to reproduce these findings are available from the corresponding author upon request.
