# Correction: Combination of ferroptosis and pyroptosis dual induction by triptolide nano-MOFs for immunotherapy of Melanoma

**DOI:** 10.1186/s12951-024-02624-z

**Published:** 2024-07-15

**Authors:** Shengmei Wang, Qiuyan Guo, Rubing Xu, Peng Lin, Guoyan Deng, Xinhua Xia

**Affiliations:** 1 School of Pharmacy, Hunan University of Chinese Medicine, Changsha, 410208 Hunan China; 2 The First Hospital of Hunan University of Chinese Medicine, Changsha, 410007 Hunan China

**Correction: Journal of Nanobiotechnology (2023) 21:383** 10.1186/s12951-023-02146-0

Following publication of the original article, the authors found errors in the quantification in Fig. 7B. “The results showed that compared with PBS or TFBF, the proportion of activated DCs (CD86^+^CD11c^+^) in TPL@TFBF tumor tissues increased from ~ 26.8 to ~ 57.3%, indicating an increased level of DCs maturation after TPL@TFBF treatment (Fig. 7A, B and Additional file 1: Fig. S13).”

Incorrect Fig. 7B
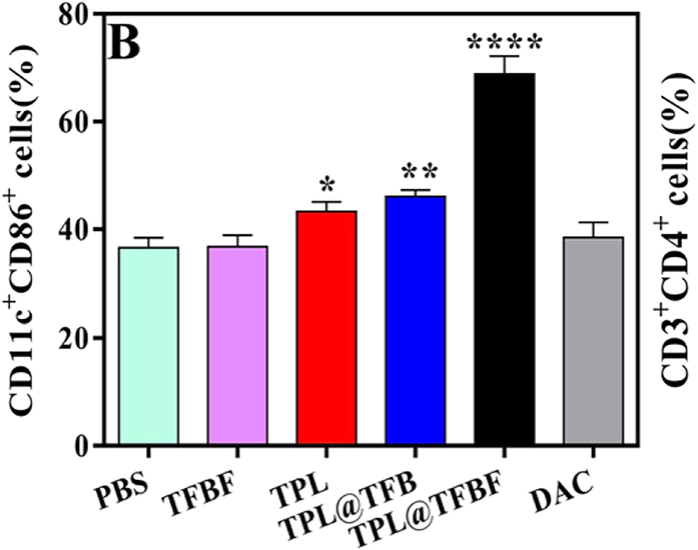


Corrected Fig. 7B
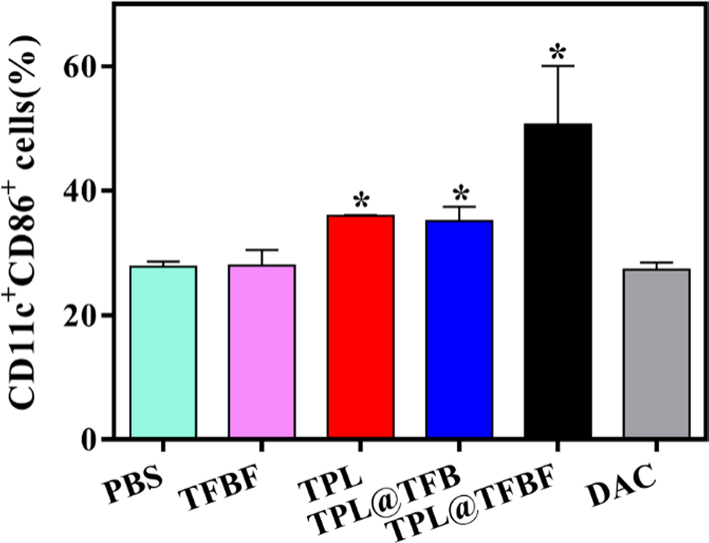


Fig. 7B Quantification of the results from (A)

The complete corrected Fig. 7 is given below.

**Fig. 7:**
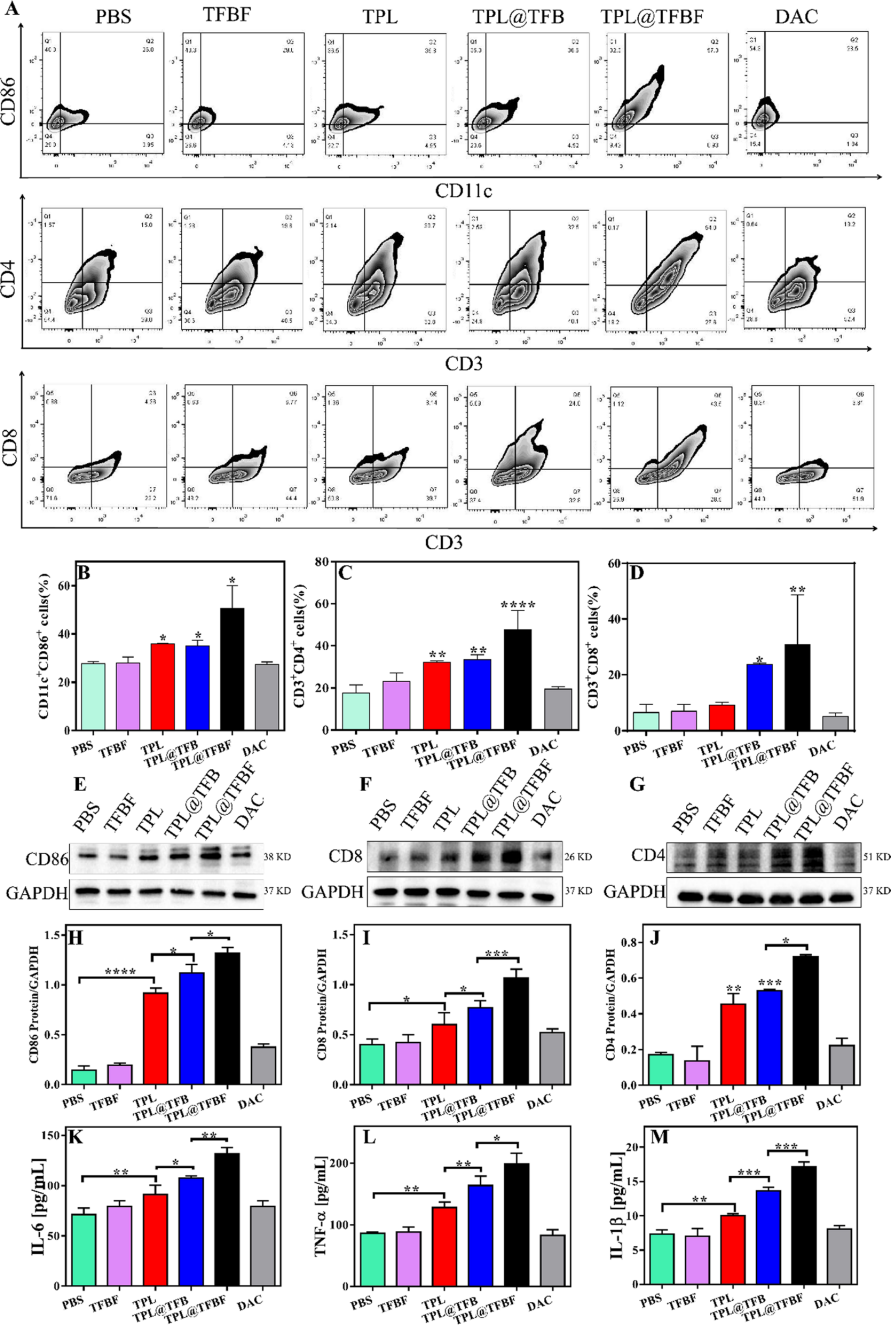
**A** Flow cytometry analysis of CD11c^+^CD86^+^ cells, CD3^+^CD4^+^ cells and CD3^+^CD8^+^ cells in tumor tissues from mice in each group after treatment with different formulations. **B**–**D** Quantification of the results from **A**. **E**–**G** Expression levels of CD86, CD8 and CD4 in tumor tissues from different groups. **H**–**J** Quantification of the results from **E**–**G**. The levels of (**K**) IL-6, (**L**) TNF-α and (**M**) IL-1β in tumor tissues from mice in different groups after different treatments. Data are expressed as the mean ± SD (n = 3). *P < 0.05, **P < 0.01, ***P < 0.001, ****P < 0.0001

The original article has been corrected.

