# Supplementary material for: Combination of ferroptosis and pyroptosis dual induction by triptolide nano-MOFs for immunotherapy of Melanoma
Source: J Nanobiotechnology. 2023 Oct 19;21:383. doi: 10.1186/s12951-023-02146-0 (PMC10585872; doi:10.1186/s12951-023-02146-0)
Supplement: Supplementary file 1 — Additional file 1: Figure S1. The particle sizes of TPL@TF and TPL@TFB. Figure S2. SDS-PAGE gel images of BSA and TPL@TFBF. Figure S3. TPL@TFBF particle size and PDI at 4℃ for 30 days. Figure S4. Determination of Fe2+ content under different treatments. Figure S5. Quantified expression of (A) Nrf2 and (B) GPX4. Data are expressed as the mean ± SD (n = 3). ns, not significant, *P<0.05. Figure S6. Quantified expression of (A) Cleaved caspase-3 and (B) GSDME-N. Data are expressed as the mean ± SD (n = 3). *P<0.05, ***P<0.001. Figure S7. The process of gating for pyroptosis cells in these cells in flow cytometry analysis. Figure S8. Hemolysis percentage of red blood cells at various concentrations of TPL@TFBF. Water treated cells were used as positive control. The negative control was 0.9% NaCl. Figure S9. Dynamically monitoring the body weight change during treatments (n = 6). Figure S10. Evaluation of the hepatotoxicity (A) and nephrotoxicity (B) of each formulation by measuring the serum levels of ALT, AST, BUN and CRE after treatments. Figure S11. H&E staining of heart, liver, spleen, lung and kidney after different treatments. Scale bar: 100 μm. Figure S12. The levels of (A) IL-6 and (B) TNF-α in serum of mice in different groups after different treatments. Figure S13. The process of gating for (A) activated DCs and (B) CD4+ and CD8+ T cells in these cells in flow cytometry analysis. Figure S14. (A) The expression of CD4 and CD8 proteins in tumor tissue after different treatments. (B) The expression of CD11c and CD80 proteins in tumor tissue after different treatments. Scale bar: 20 μm. Figure S15. Representative photographs of resected tumors. [file 12951_2023_2146_MOESM1_ESM.doc]

**Supporting Information**

**Combination of ferroptosis and pyroptosis dual induction by triptolide nano-MOFs for immunotherapy of melanoma**

Shengmei Wang^1^, Qiuyan Guo^1^, Rubing Xu^1^, Peng Lin^1^, Guoyan Deng^2^, and Xinhua Xia*^1^

^1^. School of Pharmacy, Hunan University of Chinese Medicine, Changsha, Hunan, 410208, China

^2^. The First Hospital of Hunan University of Chinese Medicine, Changsha, Hunan 410007, China

*Email: xiaxinhua001@hnucm.edu.cn


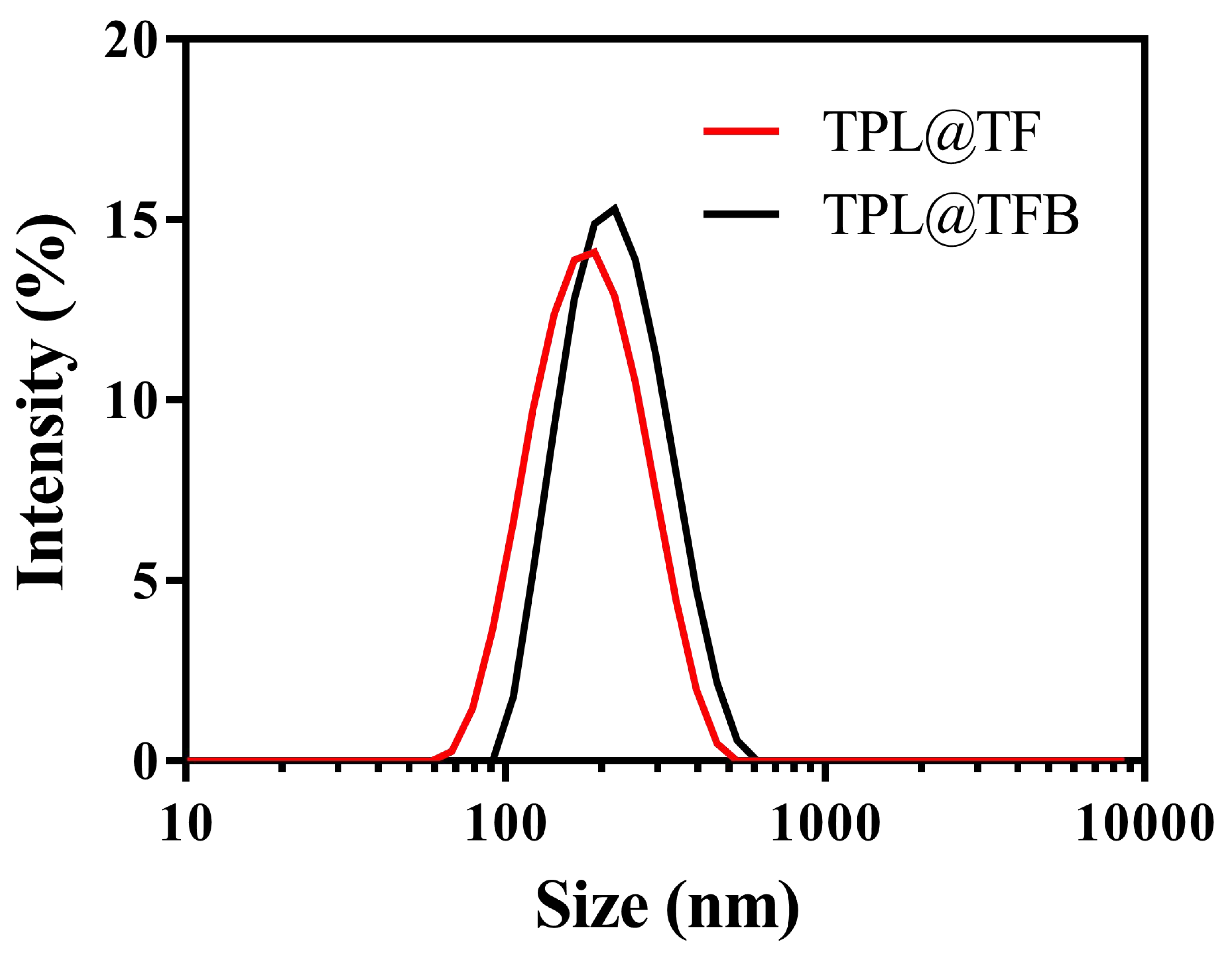


**Figure S1.** The particle sizes of TPL@TF and TPL@TFB.


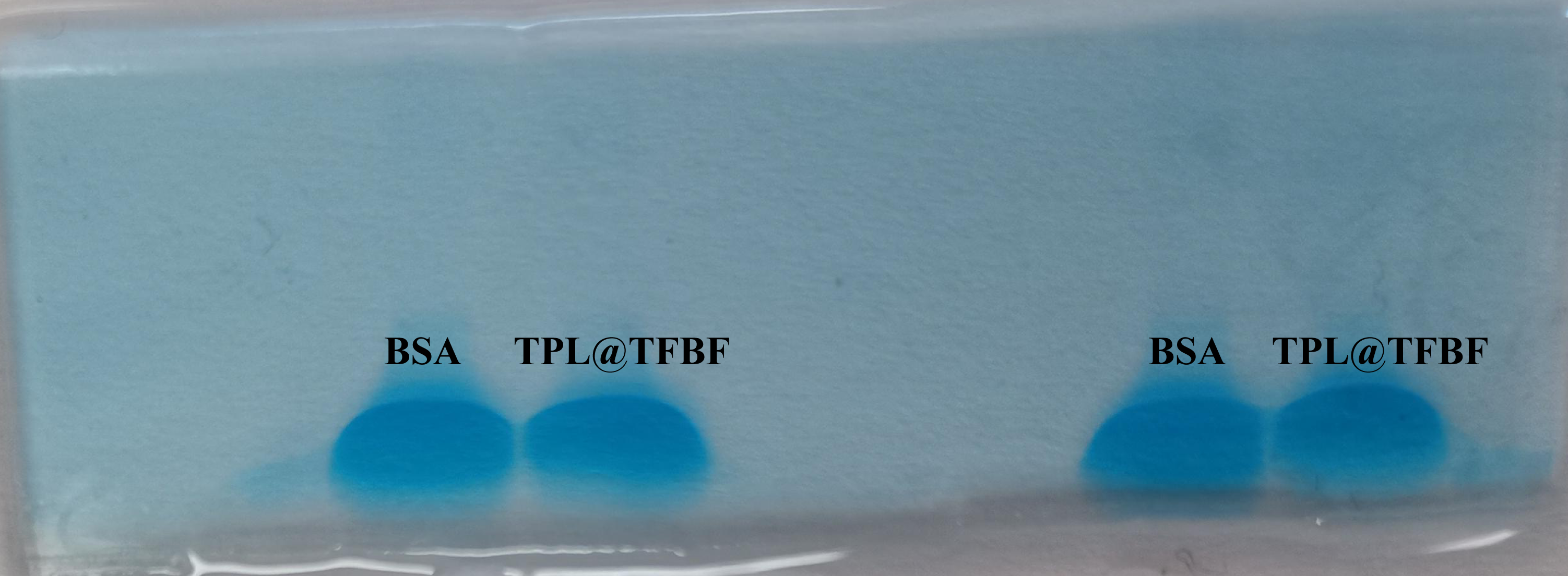


**Figure S2.** SDS-PAGE gel images of BSA and TPL@TFBF.


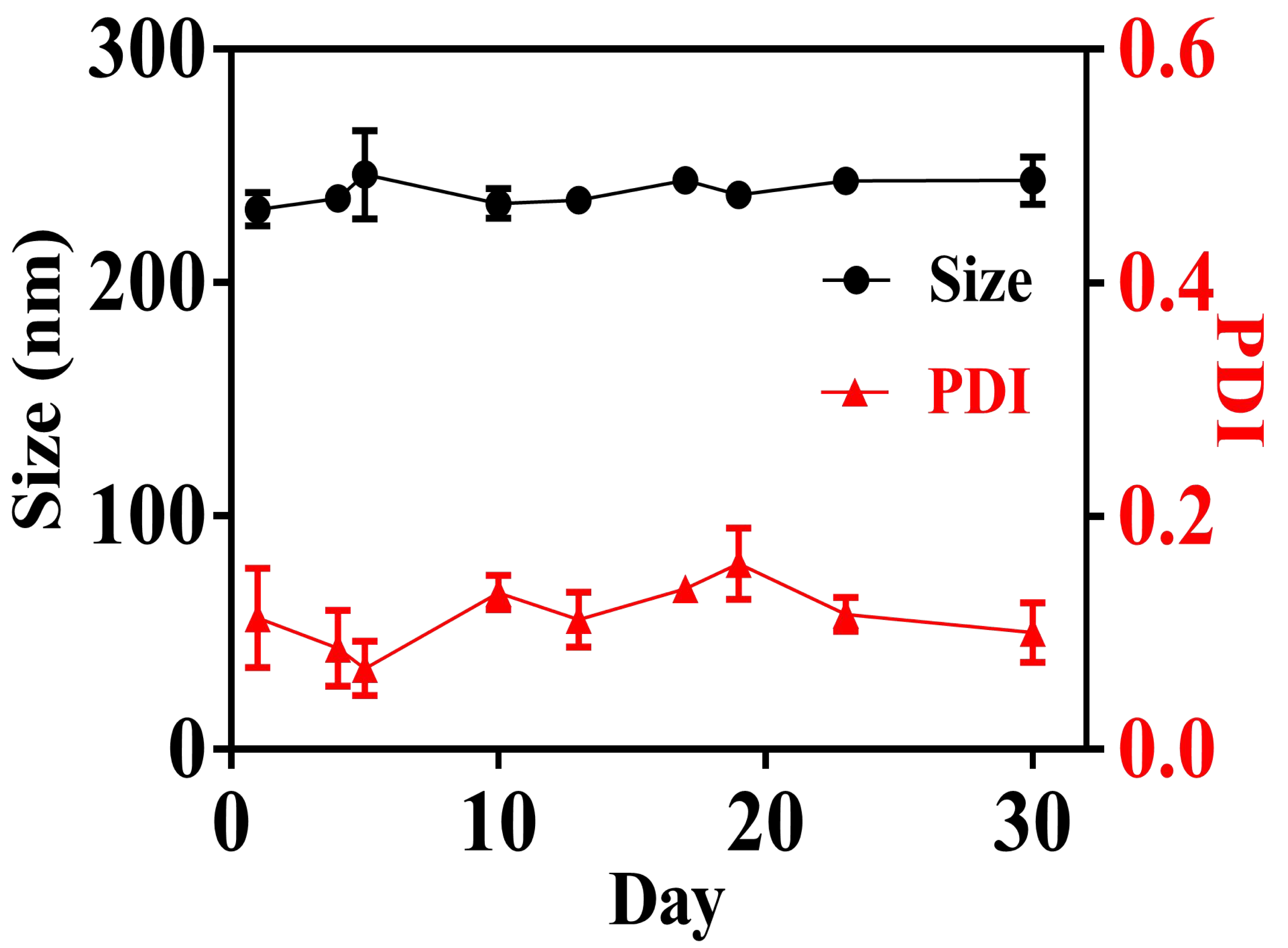


**Figure S3.** TPL@TFBF particle size and PDI at 4℃ for 30 days.


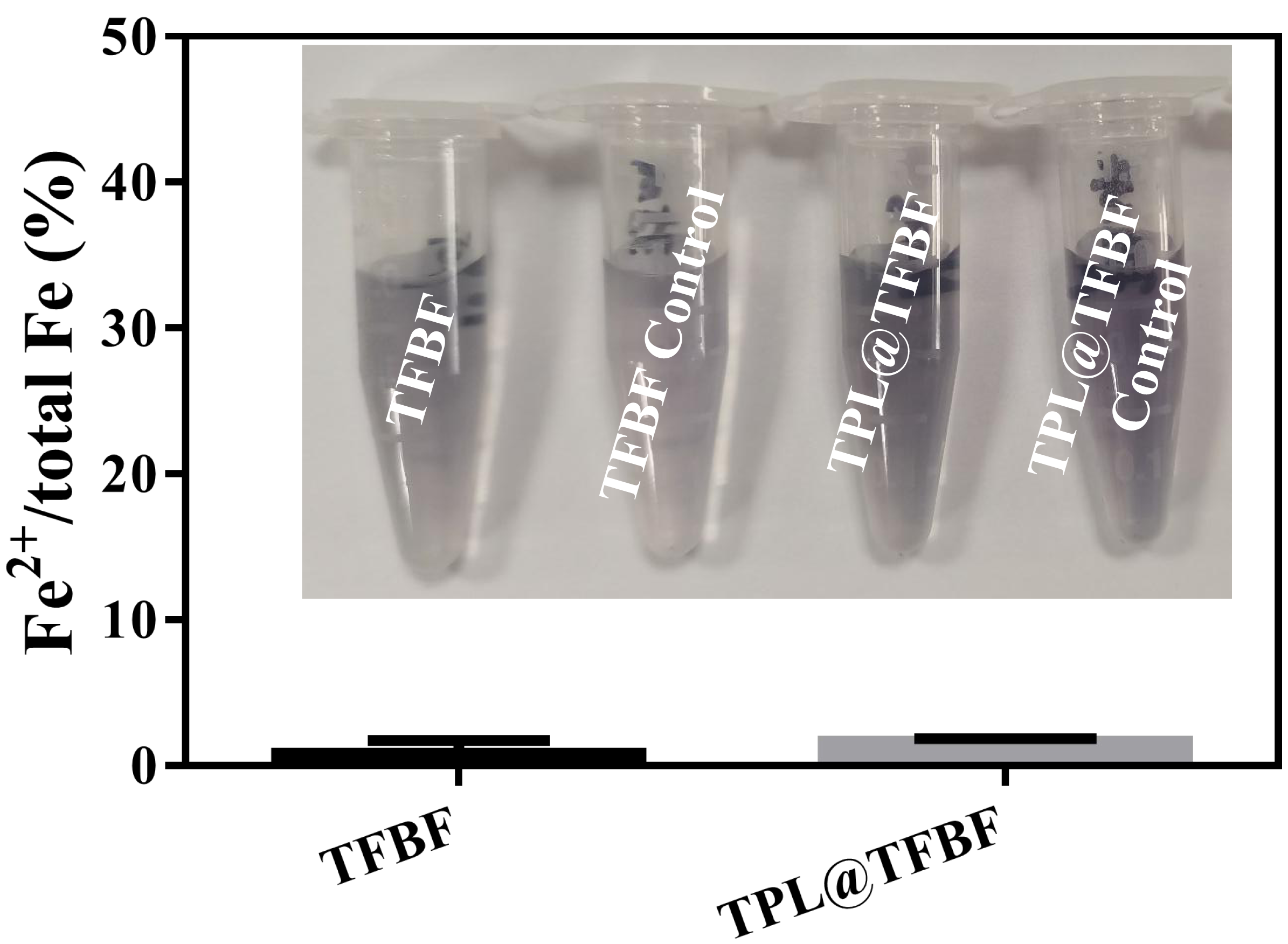


**Figure S4**. Determination of Fe^2+^ content under different treatments.


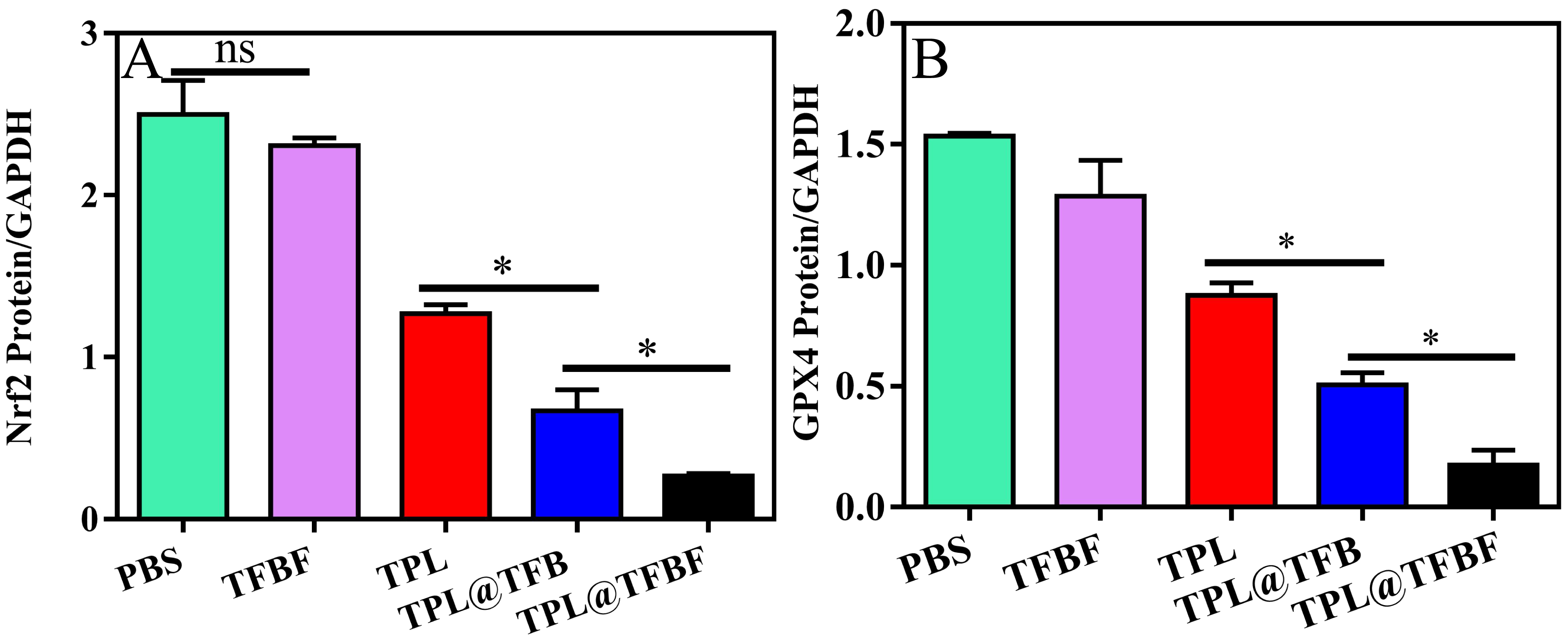


**Figure S5.** Quantified expression of (A)Nrf2 and (B) GPX4. Data are expressed as the mean ± SD (n = 3). ns, not significant, *P<0.05.


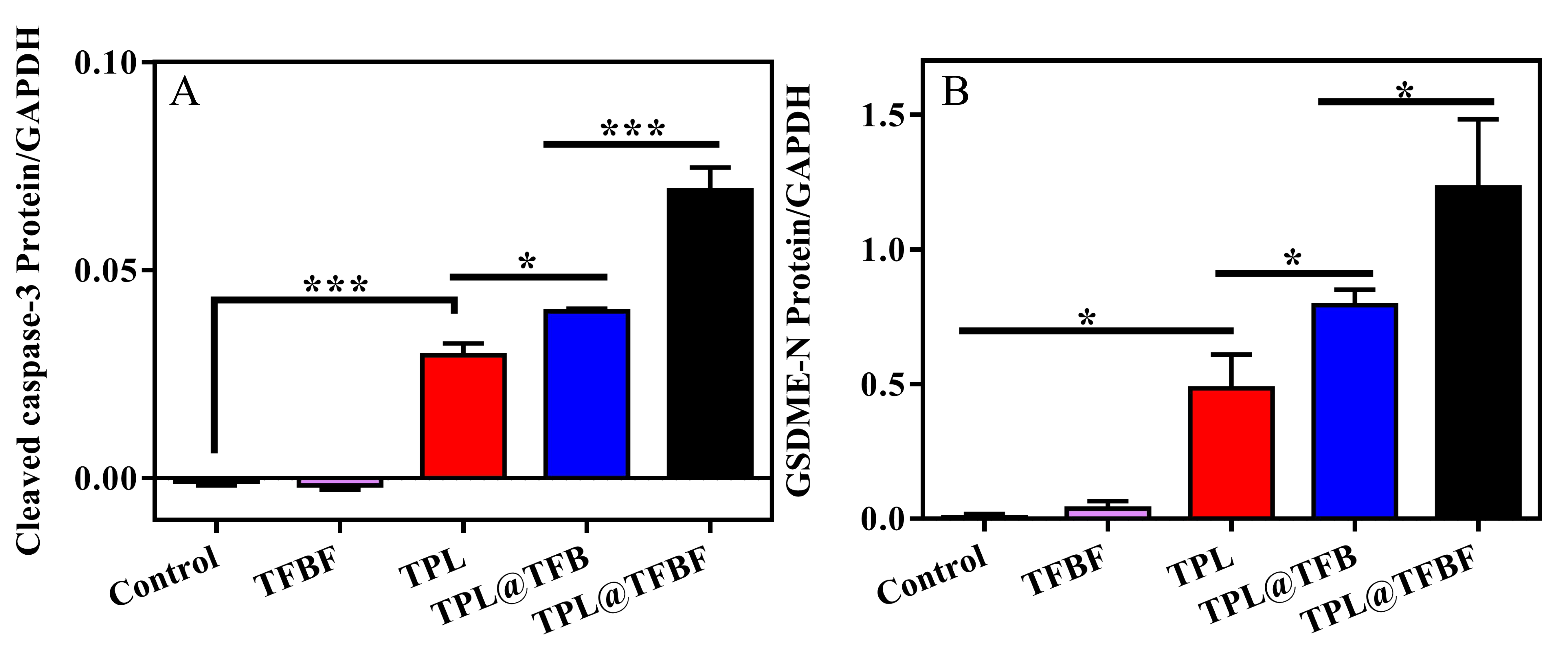


**Figure S6.** Quantified expression of (A)Cleaved caspase-3 and (B) GSDME-N. Data are expressed as the mean ± SD (n = 3). *P<0.05, ***P<0.001.


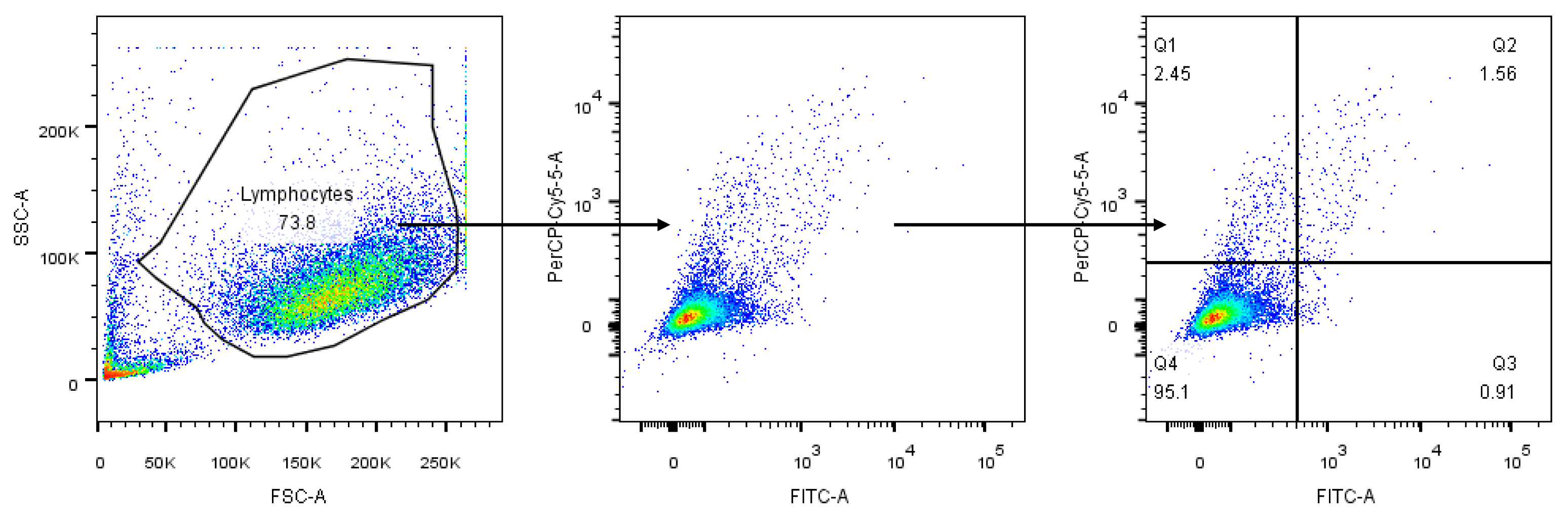


**Figure S7.** The process of gating for pyroptosis cells in these cells in flow cytometry analysis.


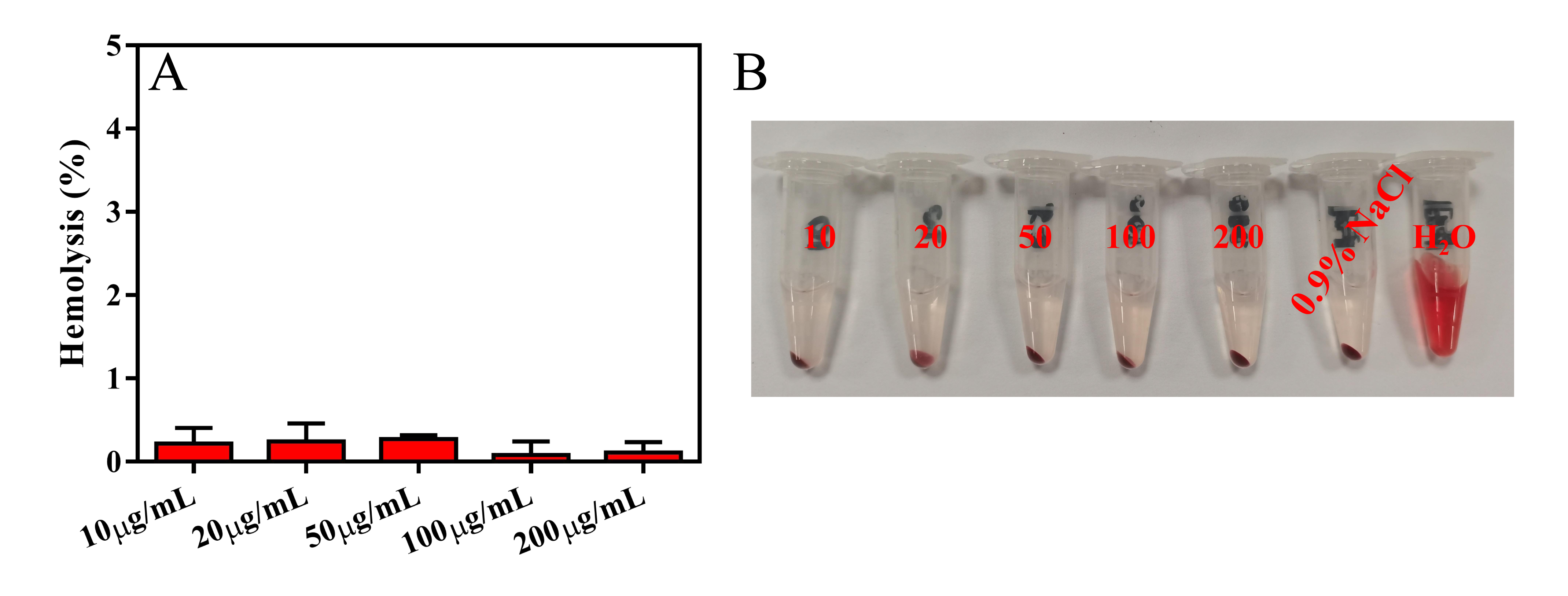


**Figure S8.** Hemolysis percentage of red blood cells at various concentrations of TPL@TFBF. Water treated cells were used as positive control. The negative control was 0.9% NaCl.

**Figure S9.** Dynamically monitoring the body weight change during treatments (n = 6).


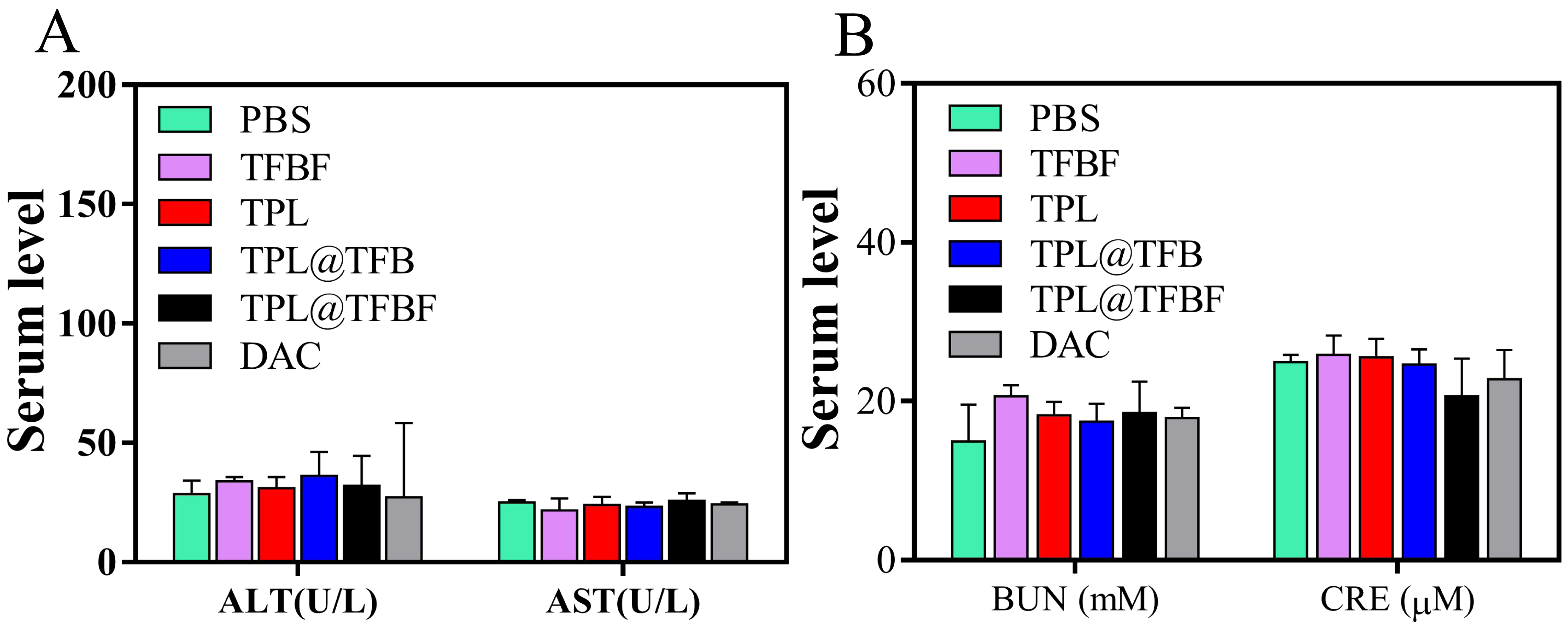


**Figure S10.** Evaluation of the hepatotoxicity (A) and nephrotoxicity (B) of each formulation by measuring the serum levels of ALT, AST, BUN and CRE after treatments.


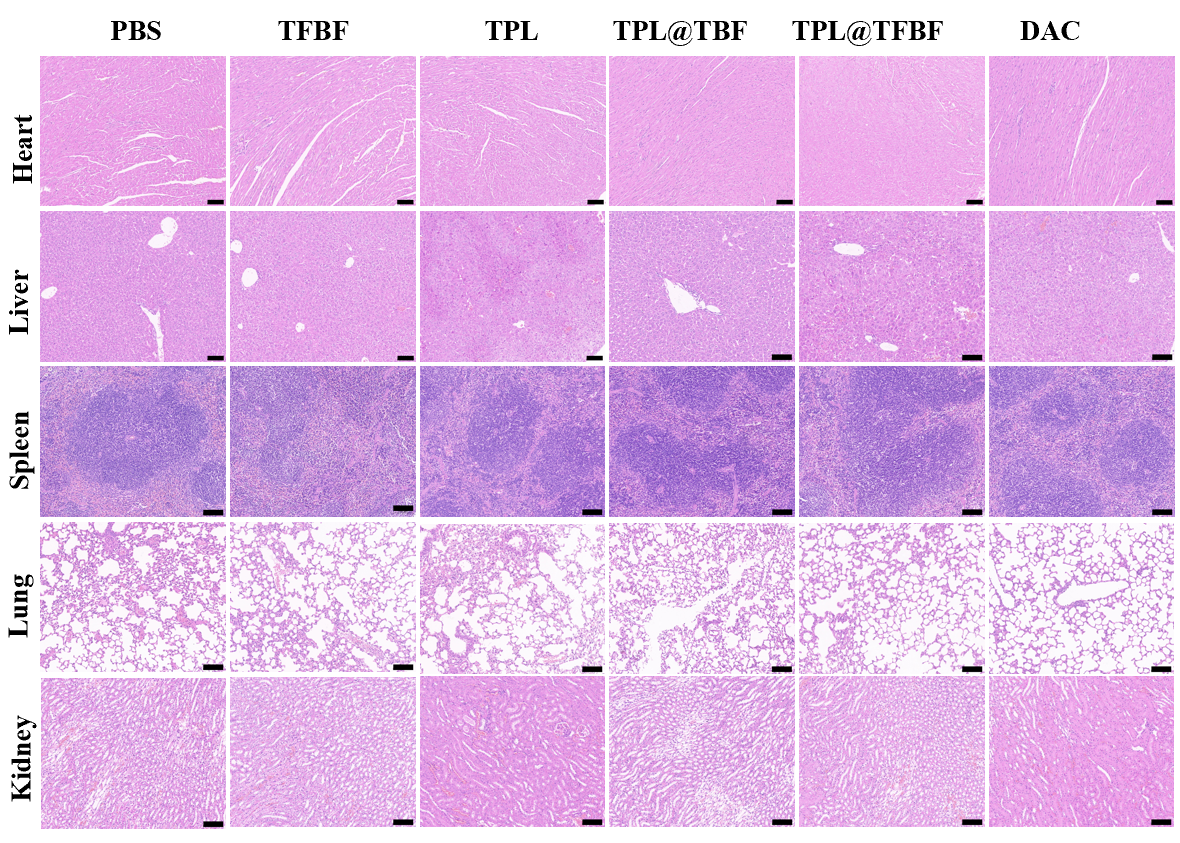


**Figure S11.** H&E staining of heart, liver, spleen, lung and kidney after different treatments. Scale bar: 100 μm.


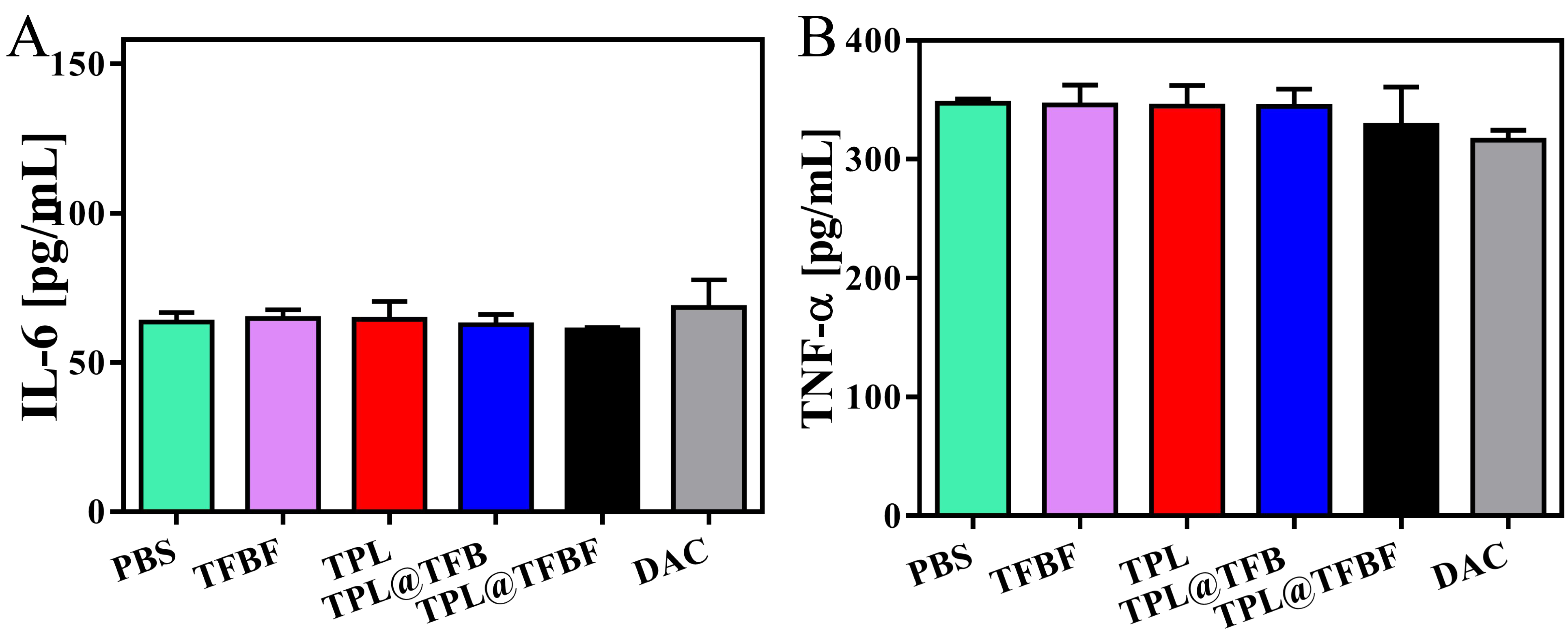


**Figure S12.** The levels of (A) IL-6 and (B) TNF-α in serum of mice in different groups after different treatments.


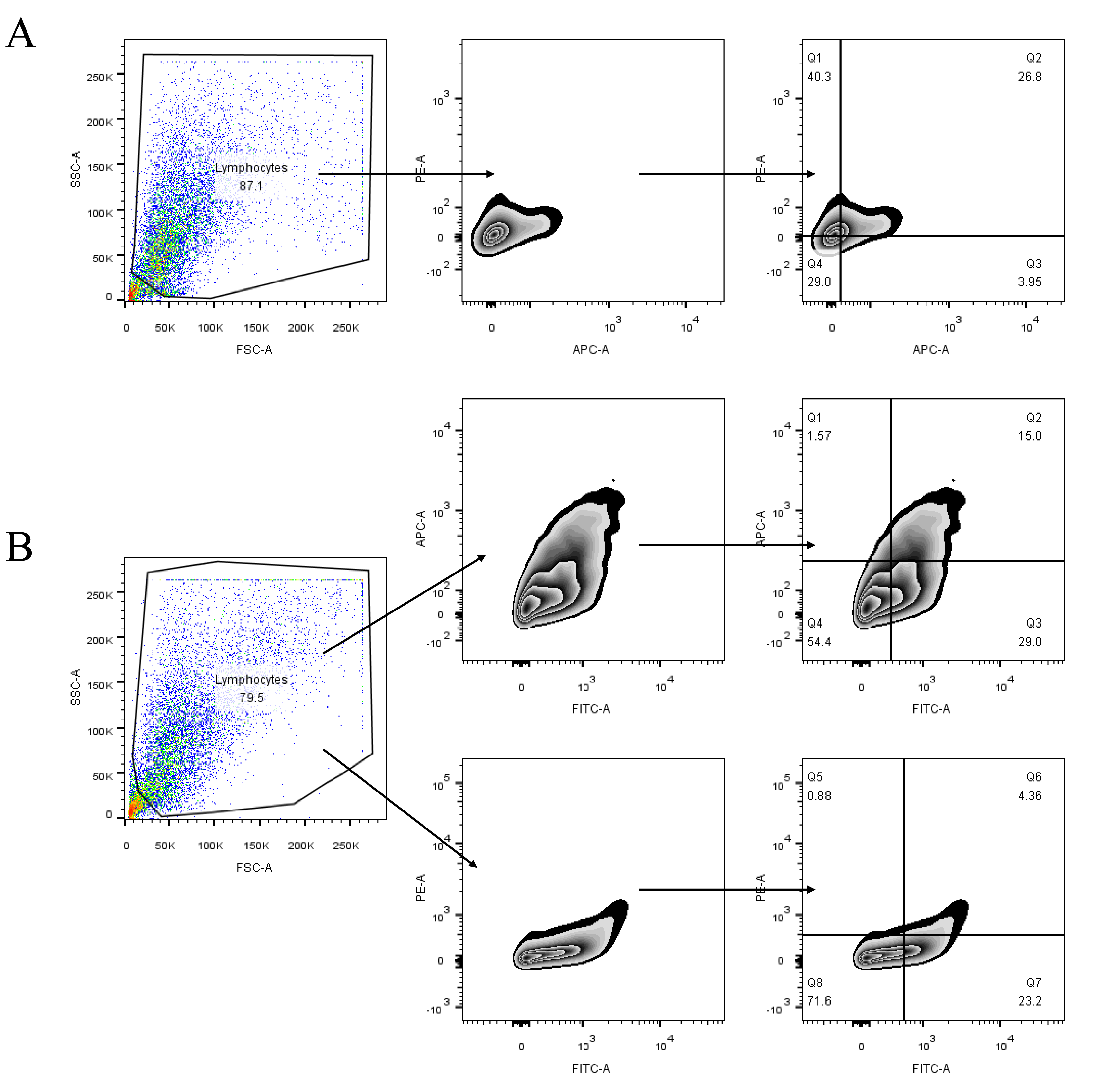


**Figure S13.** The process of gating for (A) activated DCs and (B) CD4+ and CD8+ T cells in these cells in flow cytometry analysis.

**Figure S14.** (A) The expression of CD4 and CD8 proteins in tumor tissue after different treatments. (B) The expression of CD11c and CD80 proteins in tumor tissue after different treatments. Scale bar: 20 μm.


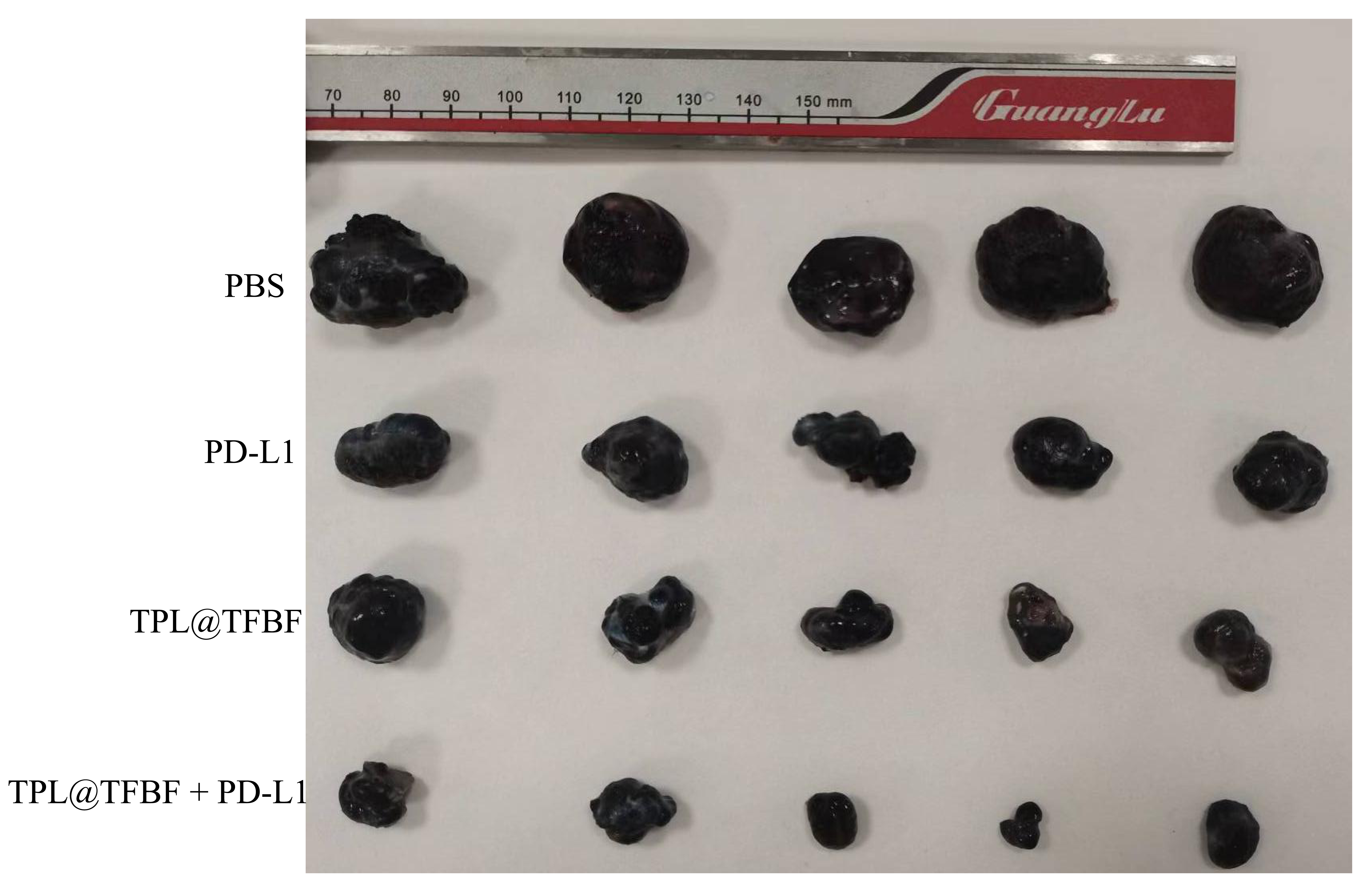


**Figure S15**. Representative photographs of resected tumors.
